# *Limosilactobacillus reuteri* administration alters the gut-brain-behavior axis in a sex-dependent manner in socially monogamous prairie voles

**DOI:** 10.3389/fmicb.2023.1015666

**Published:** 2023-02-08

**Authors:** Meghan Donovan, Calvin S. Mackey, Michael D. J. Lynch, Grayson N. Platt, Amber N. Brown, Brian K. Washburn, Darryl J. Trickey, J. Thomas Curtis, Yan Liu, Trevor C. Charles, Zuoxin Wang, Kathryn M. Jones

**Affiliations:** ^1^Department of Psychology and Program in Neuroscience, Florida State University, Tallahassee, FL, United States; ^2^Rocky Mountain Mental Illness Research Education and Clinical Center, Rocky Mountain Regional VA Medical Center, Aurora, CO, United States; ^3^Department of Physical Medicine and Rehabilitation, University of Colorado Anschutz Medical Campus, Aurora, CO, United States; ^4^Department of Biological Science, Florida State University, Tallahassee, FL, United States; ^5^Metagenom Bio Life Science Inc, Waterloo, ON, Canada; ^6^Department of Biology, University of Waterloo, Waterloo, ON, Canada; ^7^Department of Biological Science Core Facilities, Florida State University, Tallahassee, FL, United States; ^8^Department of Pharmacology and Physiology, Oklahoma State University Center for Health Sciences, Tulsa, OK, United States

**Keywords:** gut microbiome, social affiliation, anxiety-like behavior, sex difference, *Limosilactobacillus reuteri*, *Bifidobacteriaceae*, CRF-NAcc, V1aR-PVN

## Abstract

Research on the role of gut microbiota in behavior has grown dramatically. The probiotic *L. reuteri* can alter social and stress-related behaviors – yet, the underlying mechanisms remain largely unknown. Although traditional laboratory rodents provide a foundation for examining the role of *L. reuteri* on the gut-brain axis, they do not naturally display a wide variety of social behaviors. Using the highly-social, monogamous prairie vole (*Microtus ochrogaster*), we examined the effects of *L. reuteri* administration on behaviors, neurochemical marker expression, and gut-microbiome composition. Females, but not males, treated with live *L. reuteri* displayed lower levels of social affiliation compared to those treated with heat-killed *L. reuteri*. Overall, females displayed a lower level of anxiety-like behaviors than males. Live *L. reuteri*-treated females had lower expression of corticotrophin releasing factor (CRF) and CRF type-2-receptor in the nucleus accumbens, and lower vasopressin 1a-receptor in the paraventricular nucleus of the hypothalamus (PVN), but increased CRF in the PVN. There were both baseline sex differences and sex-by-treatment differences in gut microbiome composition. Live *L. reuteri* increased the abundance of several taxa, including *Enterobacteriaceae*, *Lachnospiraceae* NK4A136, and *Treponema*. Interestingly, heat-killed *L. reuteri* increased abundance of the beneficial taxa *Bifidobacteriaceae* and *Blautia*. There were significant correlations between changes in microbiota, brain neurochemical markers, and behaviors. Our data indicate that *L. reuteri* impacts gut microbiota, gut-brain axis and behaviors in a sex-specific manner in socially-monogamous prairie voles. This demonstrates the utility of the prairie vole model for further examining causal impacts of microbiome on brain and behavior.

## Introduction

The vast number of organisms that comprise the gut microbiota have recently become a research area of great focus for human health. Bidirectional connections between the gut and the brain, commonly referred to as the gut-brain axis, allow for an indirect influence of gut microbiota on brain function and resulting behaviors as well as top-down influences on microbial survival (Galland, 2014; Carabotti et al., 2015). It has been increasingly recognized that gut bacteria can influence a variety of behaviors – including both social and anxiety-related behaviors (Dinan and Cryan, 2013; Luna and Foster, 2015). In humans, high comorbidity rates exist between social/anxiety disorders and GI-related issues, further strengthening the evidence for a translational role of gut bacteria in modulating these behaviors (Mussell et al., 2008; Rosenfeld, 2015). An evolutionary hypothesis has been proposed for gut microbiota increasing the social nature of humans, suggesting a fundamentally critical role for proper social development (Sherwin et al., 2019). The intake of probiotics, or live bacteria deemed beneficial for human health, has increased dramatically in recent years. Given the intricate connections between gut microbes and the brain, a full understanding of how probiotics alter the gut-brain axis and resulting behaviors is critical. Yet, the underlying mechanisms remain much unknown.

Animal research on the gut-brain axis has focused on revealing underlying mechanisms of probiotic impacts on the brain and behavior. For example, intake of probiotics can alter both social and anxiety-like behaviors as well as brain neurochemical systems, such as oxytocin (OT) and corticotropin releasing factor (CRF), in several animal models (O'Sullivan et al., 2011; Buffington et al., 2016; Liu et al., 2016; Wang et al., 2016). Mice raised in a germ-free environment display atypical social behavior, which can be restored with bacterial reinoculation (Sudo et al., 2004; Desbonnet et al., 2013). Microbe-free mice also show deficits in their stress response system and changes in anxiety-like behaviors (Sudo et al., 2004; Neufeld et al., 2011; De Palma et al., 2017). These microbiota-specific effects on the brain and behavior also differ in males and females throughout development and adulthood (Clarke et al., 2012; Jašarević et al., 2016). *L. reuteri* (formerly *L. reuteri*), a common probiotic, has become a focus in some studies. Administration of live, but not heat-killed, *L. reuteri* upregulates OT in the paraventricular nucleus of the hypothalamus (PVN) and restores prosocial behavior in mice bred from mothers fed high-fat diets (Buffington et al., 2016). Administration of *L. reuteri* also leads to wound healing improvement, which is associated with increased systemic OT levels and PVN OT expression (Varian et al., 2017). These data set up a strong foundation for focusing on *L. reuteri’s* influence on social behaviors and underlying neurochemical circuits. However, it should be noted that traditional laboratory rodents do not display many naturally complex social behaviors. Furthermore, although genetically similar rodent models provide a great way to control and determine causal mechanisms, they fail to encapsulate genetic complexities and individual variation. Moreover, the majority of research done in traditional rodents has also primarily focused on manipulations (i.e., maternal immune activation, valproic acid, etc.) to examine microbial impacts on animal-modeled disorders (Nithianantharajah et al., 2017; Needham et al., 2018). Exploring the impacts of microbial manipulation *via* probiotic intake on natural social behaviors and the underlying neurochemical circuits in an optimal animal model is needed.

The socially monogamous prairie vole (*Microtus ochrogaster*), a rodent commonly found in grasslands of the United States, is a well-established animal model for examining the neurobiology of social behavior due to their natural behaviors that are easily tested in laboratory settings (Young et al., 2011a). Prairie voles reliably display a wide array of social behaviors similar to humans, including the formation of socially monogamous pair bonds, biparental care, and high levels of social affiliation (Tabbaa et al., 2017). The underlying neurochemical circuits responsible for social behavior have been well documented in previous vole studies (Young et al., 2011a), allowing for a unique opportunity to examine how these natural circuits may be altered by probiotic intake. Further, commensal bacteria, like *L. reuteri*, are naturally present in their gut microbiome, further justifying their translational use for the study of microbial influences on social behavior and underlying neurochemical circuits (Assefa et al., 2015). Prairie voles are also well-established for studying anxiety-like behaviors and the stress response, making them an ideal model for concurrently assessing the effects of *L. reuteri* on both social and stress circuits in the brain (Smith et al., 2013; Smith and Wang, 2014). Laboratory prairie voles are captive bred, which allows for better maintenance of genetic variation and individual differences. We have successfully examined the gut microbiome in prairie voles *via* metagenomic sequencing and found that it contains novel, dominant bacterial strains (Donovan et al., 2020a). Thus, a study on how probiotics, such as *L. reuteri*, alter social and anxiety-like behaviors, neurochemical expression in the brain, and the gut microbiota in male and female prairie voles is well-justified. Taken together, the prairie vole model presents a unique opportunity for reducing the gap between rodent and human gut microbiome research.

As prairie vole neurochemical social circuits in the brain have been studied extensively, there is already a strong foundation upon which to focus in the current study. Key neurochemicals known to be involved in the prairie vole social neurochemical circuits, such as OT, arginine vasopressin (AVP), and CRF, can be altered by gut microbiota (Holzer and Farzi, 2014; Fields et al., 2017). In the present study, we examined the effects of *L. reuteri* administration on anxiety-like and social affiliation behaviors in male and female prairie voles. We hypothesized that live bacterial administration would alter behavior, these differences in behavior would be correlated with alterations in brain OT expression, and these specific effects may differ between male and female prairie voles.

## Materials and methods

### Subjects

Subjects were male and female prairie voles (*M. ochrogaster*) captive-bred at Florida State University. Voles were weaned on postnatal day 21 and housed in Plexiglas cages (20 × 25 × 45 cm) with a same-sex conspecific. Cages contained cedar chip bedding with food and water provided *ad libitum*. Subjects were kept at 20°C under a 14: 10 h light: dark cycle (lights on at 0700). At the time of testing, subjects had reached adulthood (> 90 days) and were sexually naïve. Behavioral testing was performed after 4 weeks of *L. reuteri* treatment. Pre- and post-treatment weights and water consumption are shown in Supplementary Table S1. All procedures were approved by the Institutional Animal Care and Use Committee at Florida State University and were in accordance with the guidelines set forth by the National Institutes of Health.

### *L. reuteri* growth and collection

*L. reuteri* strain MM4-1A (obtained from the American Type Culture Collection [ATCC] strain PTA-6475) was grown anaerobically in a Gas-Pak system (Becton Dickinson, Franklin Lakes, NJ, United States) in MRS medium (Hardy Diagnostics, Santa Maria, CA, United States) at 37°C for 24 h. Under sterile conditions, cells were concentrated by centrifugation, washed and resuspended in phosphate-buffered saline (PBS)/10% glycerol at an optical density at 600 nm (OD_600_) of 80–100, and immediately frozen in 2 mL aliquots at −80°C. At least 1 frozen aliquot from each live preparation was thawed and plated to MRS agar (BD-Difco, Sparks, MD, United States) to confirm live *L. reuteri* at a concentration of 4.0E09-1.3E10 colony-forming units (cfu) per aliquot. Aliquots from 2 separate batches of *L. reuteri* were used to construct 16S rRNA gene V3-V4 amplicon libraries. These were sequenced and analyzed as described below and found to be composed of only *L. reuteri* sequences (Supplementary Figure S1). Heat-killed (HK) aliquots of *L. reuteri* were prepared as above, but were killed by incubation at 80°C for 2–3 h and re-frozen. At least one aliquot of killed cells for each batch was tested for complete killing by plating to MRS agar. Aliquots were stored at −80°C until the time of use.

### *L. reuteri* administration

We chose to study the difference between HK-*L. reuteri* and live-*L. reuteri* treatment because one of our main goals was to compare the effects of level of host *L. reuteri* colonization with the effects of ingestion of the same biomass of *L. reuteri* cellular metabolites and macromolecules. Each subject (*n* = 8 per sex per treatment, an effective group size evidenced by previous vole studies on neurochemical regulation of behavior) was housed with a same sex cagemate that received the same treatment. Voles from different cages were randomly assigned to receive drinking water with either live *L. reuteri* or HK (control) *L. reuteri*. *L. reuteri* aliquots were administered each morning for 28 days in ddH_2_O. The dose (final concentration of live *L. reuteri* 4E^+07^–1.3E^+08^ cfu/mL in drinking water) used in the current study was adapted from a previous study examining *L. reuteri* effects on social behavior in mice (Buffington et al., 2016). The intake of water per cage was recorded for the entire treatment duration. All subjects were weighed pre- and post-bacterial treatment.

### Behavioral testing

The open field (OF) test has been established in our previous study in prairie voles (Lieberwirth et al., 2013). The open field arena consists of a plastic box [56 × 56 × 20 (H) cm] with a lined floor divided into 16 squares (14 × 14 cm). In the morning on day 1 of behavioral testing, subjects were placed individually in the center square of the arena and allowed to freely roam for 10 min. All behaviors were recorded using Active Webcam software and were quantified by an observer blind to treatment *via* J-Watcher.^1^ Behaviorsquantified included frequency and duration in center, periphery, and corner squares. Total square crosses were also quantified to measure overall locomotor activity.

The social affiliation (SA) test has also been established in our previous study (Pan et al., 2009). The testing apparatus consists of two polycarbonate chambers [13 × 18 × 29 (H) cm] both containing clean cedar chip bedding along with food and water that were connected by a hollow tube (7.5 × 16 cm). A same sex, unfamiliar stimulus animal at a similar age and size as the subject was loosely tethered in one chamber, and the subject was placed into the empty chamber to freely roam the SA apparatus for 1 h. Subjects underwent the SA test in the afternoon on day 1 of behavioral testing. Behaviors were recorded using Active Webcam software and the first 20 min were subsequently quantified by an observer blind to treatment *via* J-Watcher. Behaviors quantified included frequency and duration in the conspecific cage, empty cage, and connecting tube. Additional behavioral quantifications included duration and frequency of specific behaviors (see Supplementary Table S2).

The elevated plus maze (EPM) test has been established and validated in our previous vole study (Smith and Wang, 2014). Briefly, the apparatus is elevated 45 cm off the ground and consists of two open (35 × 6.5 cm) and two closed arms [35 × 5 × 15 (H) cm] that cross in the middle. All subjects underwent EPM testing in the morning on day 2 of behavioral testing. Subjects were placed onto the center of the maze facing an open arm. Subject’s behaviors were recorded for 5 min using Active Webcam software and were subsequently quantified by an observer blind to treatment *via* J-Watcher. Behaviors quantified included the duration and frequency in the open arms, closed arms, and the center of the EPM. The percentage of time in the open arms and locomotor activity (as indicated by total crosses) were also calculated. During each behavioral test, subjects treated with either live- or HK-*L. reuteri* were randomly processed.

### Brain tissue preparation

All subjects were rapidly decapitated immediately after the post-treatment stool collection. Brains were immediately extracted and placed on dry ice. All brains were stored at −80°C until processing. Tissue punches were collected from coronal sections (200 μm thick) from the prefrontal cortex (PFC), amygdala (AMY), nucleus accumbens (NAcc), paraventricular nucleus of the hypothalamus (PVN), and ventral tegmental area (VTA) (four sections per region per subject). Tissue punches were stored at −80°C until subsequent protein extraction.

### Protein extraction

Protein extractions were performed using established methods (Smith and Wang, 2014). Briefly, protein was extracted from tissue punches using Tri-Reagent according to the manufacturer instructions (Molecular Research Center, Cincinnati, OH, United States). Protein was stored at −80°C until Western Blotting. Protein extractions from all sampling areas were measured using a DC protein assay (Bio-Rad) to determine total protein concentrations.

### Western blotting

Western blotting procedures were performed using our established methods (Smith and Wang, 2014). Briefly, 15 μg of protein were mixed with loading buffer and loaded into 12.5% sodium dodecyl sulfur (SDS) polyacrylamide gels (Bio-Rad) for electrophoresis. Proteins were run on gels at 75 volts (V) for approximately 30 min and thereafter at 200 V for 80 min under refrigeration. Proteins were then transferred to PVDF membrane in Tris-glycine buffer with 20% methanol at 4°C. Membranes were subsequently blocked in Tris buffered saline (TBS) with 5% nonfat dry milk or SuperBlock T20 (TBS) (Thermo Scientific, Rockford, IL, United States). Membranes were incubated for 1–2 days with one of the following primary antibodies: goat anti-oxytocin receptor (OTR) (1: 1 K, Santa Cruz), goat anti-vasopressin receptor (V1aR) (1: 1 K, Santa Cruz), rabbit anti-corticotropin releasing factor (CRF) (1:500, ProteinTech), rabbit anti-corticotropin releasing factor receptor 1 (CRFR1) (1:500, Novus Biologicals), rabbit anti-corticotropin releasing factor receptor 2 (CRFR2) (1:500, Novus Biologicals), goat anti-vasopressin receptor (V1aR) (1:1 K, Aviva), rabbit anti-glyceraldehyde 3-phosphate dehydrogenase (GAPDH) (1:1 K, Santa Cruz). Thereafter, membranes were washed in TBS and incubated for 2 h in the respective horseradish peroxidase (HRP) conjugated secondary antibody (1:10 K, Santa Cruz). Membranes were washed again in TBS for 1 h. All membranes were then placed in chemiluminescence HRP substrate (SuperSignal West Dura Extended Duration Substrate, Thermo Fisher Scientific) for 10 min. Bands were visualized on the ChemiDoc MP System (Bio-Rad). Western band quantification was done through ImageLab software. All markers were normalized using GAPDH as a loading control.

### Data analyses

Experimenters were blind to group allocation during behavioral testing and subsequent analyses. All behavioral data were blindly scored *via* J-Watcher and analyzed by two-way ANOVA (SEX X TREATMENT) using IBM SPSS Statistics 19 software (SPSS, Inc.). Significant sex-by-treatment interactions were further examined by the Student–Newman–Keuls (SNK) *post hoc* test. Anxiety-like behaviors for the EPM and OF tests were also analyzed by averaging the z-score conversions for the percentage of time spent in the open arms of the EPM test and duration of time in the center of the OF test. Western blot results were analyzed *via* one-way ANOVA using SPSS separately for male and female subjects, as different antibodies were used due to discontinuance during experimental processing. The density of Western bands were analyzed by Image Lab software (Bio-Rad), normalized to the reference protein (GAPDH), and presented as the percent change from controls. Due to corrupt video files and typical loss of useable tissue, some subjects were not included in analyses. Significant outliers were removed from analyses. Data are shown as mean ± SEM. Values for each neurochemical marker Western band density relative to the control GAPDH band density are shown in Supplementary Table S3 (females) and Supplementary Table S4 (males).

### Stool sample collection and DNA extraction

Stool samples were collected using our established procedures (Donovan et al., 2020b). All samples were stored at −80°C until further processing. Pre-treatment collections occurred 1 day before *L. reuteri* treatment, and post-treatment collections occurred in the afternoon on day 2 of behavioral testing. DNA was prepared from −80°C-frozen stool samples using the MoBio/QiaAmp PowerFecal DNA kit, according to manufacturer’s instructions (Qiagen-USA, Germantown, MD, United States). Isolated DNA was quantified by absorbance at 260 nm on a Nanodrop spectrophotometer and by fluorescence using the dsDNA HS DNA Assay on a Qubit fluorometer (both instruments by ThermoFisher, Waltham, MA, United States).

### qPCR quantification of DNA from members of the *Lactobacillaceae* family in stool

qPCR was performed using an Applied Biosystems Quantstudio7 Flex 384-well Real-Time PCR System, with Invitrogen SYBR Green PCR Master Mix (Product number 4309155) and gene-specific primers at 0.5 μM each in an 8 μL reaction. Each reaction contained 3.3 ng bacterial DNA as determined by Qubit. Cycling conditions were: 95°C for 10 min, followed by 40 cycles of 95°C for 15 s, 53°C for 30 s and 68°C for 30 s. 60–95°C melt curve analysis following PCR was performed using default settings. Gene-specific primers were: 16S rRNA gene V3-V4 variable region amplicon primers (V3-V4)(Klindworth et al., 2013) (forward S-D-Bact-0341-b-S-17 and reverse S-D-Bact-0785-a-A-21); *Lactobacillus/Leuconostoc/Pediococcus*-specific 16S rRNA gene primers, F-lacto05 5 ′AGCAGTAGGGAATCTTCCA, R-Lacto04 5′CGCCACTGGTGTTCYTCCATATA (Mayeur et al., 2013) (hereafter referred to as ‘*Lactobacillus*’); *L. reuteri*-specific 16S-23S rRNA gene spacer primers, F- L.reuter07 5′CGTACGCACTGGCCCAA; R- L.reuter06 5′TATGCGGTATTAGCATCTGTTTCC (Mayeur et al., 2013); L. gasseri/johnsonii-specific 16S rRNA gene primers, F-L.gasse03 5′AGTCGAGCGAGCTAGCCTAGATG; R-L.gasse02 5′AGCATCTGTTTCCAGGTGTTATCC (Mayeur et al., 2013). All qPCR analysis was by ΔCt of specific primers normalized to ΔCt of 16S rRNA gene V3-V4 region (ΔΔCt).

### 16S rRNA gene amplicon library construction and sequencing

16S rRNA gene V3-V4 variable region amplicon libraries were prepared according to Illumina’s 16S Metagenomic Sequencing Library Preparation reference guide (part#15044223B).^2^ V3-V4 region amplicon primers (Klindworth et al., 2013) were modified to include a heterogeneity spacer containing 0–3 random nucleotides between the 16S rRNA gene sequence and the adaptor sequence, in order to create more sequence diversity and eliminate the need for PhiX spike-in (Fadrosh et al., 2014). Libraries tagged with different bar-codes were pooled, quantified with the KAPA Library Quantification Kit for Illumina platforms (Roche, Basel, Switzerland), and sequenced using Illumina MiSeq v3 600 cycle reagents, according to manufacturer’s instructions. All sequence and sample data is deposited at NCBI within BioProject PRJNA674340. Accession numbers for individual animal samples and raw reads can be found in Supplementary Table S5. No-template controls were included on each sequencing library plate, and all yielded too little amplicon DNA to be detected by Qubit fluorimetric DNA quantification (Qubit, ThermoFisher Scientific). An aliquot of the ABRF-MGRG 10-Strain Even Mix Genomic Material mock community (MSA-3001, American Type Culture Collection) was included on each sequencing library plate (Supplementary Table S6). The composition analysis of the mock community sequencing is shown in Supplementary Figure S1.

### 16S rRNA gene sequence analyses

Raw paired-end 16S rRNA gene sequences had their first 5 base pairs trimmed and were truncated to a length of 280 based on quality score summaries visualized with Multi-QC (Ewels et al., 2016). Sequences were then imported into QIIME2 (Bolyen et al., 2019), checked with the cutadapt plugin (Martin, 2011) and then filtered, denoised and clustered into ASVs using the Dada2 wrapper (Callahan et al., 2016), using the default maximum expected error filter of 2. Clustered sequences were then classified in the QIIME2 environment using a naïve-Bayes classifier trained off the 16S SILVA rRNA gene 97% identity database (Quast et al., 2013). Representative sequences, classified at a confidence threshold of 0.7, were further summarized into feature count tables and exported to allow transposition and manipulation into treatment group subsets. Further analysis was then performed outside the QIIME2 environment.

### Whole genome shotgun library construction, sequencing, and analysis pipeline

Genomic DNA was sheared using a Covaris E220 focused-ultrasonicator. Libraries were prepared using the NEBNext^®^ Ultra^™^ II DNA Library Prep Kit for Illumina (New England Biolabs, Ipswich, MA, United States), following the manufacturer’s protocol. 500 ng total DNA was found to be sufficient for high-yield libraries. For the 32 samples from the 16 *L. reuteri*-treated females, the average library fragment size was 885 bp. Sequencing library and library pools quantified by KAPA assay. The shotgun metagenome sequencing of 32 sample libraries from the 16 *L. reuteri*-treated females was performed on an Illumina HiSeq 2500 sequencer in the Florida State College of Medicine Translational Science Laboratory. Paired-end 250-base sequence reads were generated in the cBOT Rapid Run protocol. The Metagenome-ATLAS (Automatic Tool for Local Assembly Structures) pipeline was used for whole-genome shotgun sequence assembly, annotation and genomic binning (Kieser et al., 2019). Further analysis steps were common to the 16S rRNA gene analysis pipeline and are described below.

### Differential representation analyses of taxa in both the WGS and 16S rRNA gene datasets

The feature count table was normalized using the DESeq2 (Love et al., 2014) R package to extract the mean log ratio size factors which the counts were divided against. The normalized count table was then subset into groups to allow comparison of the female, male, pretreatment and posttreatment groups. Subset count tables were then analyzed using the DESeq2 R package’s Differential Expression wrapper (Love et al., 2014), and using the Benjamini-Hochberg False Discovery Rate procedure to correct for multiple comparisons (Benjamini and Hochberg, 1995). The false positive correction used the adjusted *p* value cutoff of 0.1. A separate method designated ‘Vole-by-Vole’ (VBV) on the DESeq2-normalized count tables was also performed using Microsoft Excel. Briefly, in this method, differences in representation of each microbial taxon were determined for each individual animal pre- to post-*L. reuteri* treatment. For both methods, a filtering criteria of an average log2 fold change of ≥|0.693| was used to select taxa of interest. The VBV pairwise method takes into account the individual variability of animals by requiring that each taxon have a supermajority of animals in the group meet this log2fc criterion. In addition, the method requires that the average log2 fold change of all animals in the group be ≥|1.25| in the same direction. Supermajority cutoffs were 5/8 for each sex-specific probiotic treatment group.

Mean relative abundance of taxa in the different sample groups was calculated using the phyloseq R package (McMurdie and Holmes, 2013). First, samples were merged into the treatment groups of interest using phyloseq::merge_samples and counts were transformed into relative abundance by recursively dividing the raw counts by the total sum of counts for each treatment group through phyloseq::transform_sample_counts. Then, taxonomic hits were agglomerated into the taxa level desired using phyloseq::tax_glom. The results were plotted using the ggplot2 package after cleaning the data by pruning all taxonomic hits with a total sum of less than 0.01 (Wickham, 2011). Abundance quartiles were determined from average pre-manipulation raw taxa counts.

### Microbial diversity analyses

Both the alpha and beta diversity metric analysis were performed using the phyloseq R package (McMurdie and Holmes, 2013) supplemented with both the vegan (Oksanen et al., 2013) and ape (Paradis et al., 2004) packages. The Chao1 (Chao, 1984) estimator for richness, the Shannon (Shannon and Weaver, 1949) effective number of species (ENS), and the Inverse Simpson (Simpson, 1949) diversity metrics were used for alpha diversity comparisons. Significant differences in sex and in probiotic treatment were defined as those with a *p* value ≤0.05 in two-tailed Mann–Whitney *U* tests for two independent samples. Significant differences for pre- vs. post-treatment were defined as those with a *p* value ≤0.05 in two-tailed Wilcoxon matched-pairs signed rank tests.

Multi-dimensional scaling analysis was performed in phyloseq and plotted with the ggplot2 package (Wickham, 2011) to visualize the beta-diversity using several methods: Bray–Curtis dissimilarity was used to quantify the difference in species abundance without regard to phylogenetic relationships between taxa (Bray and Curtis, 1957). Beta diversity between communities was also determined using unweighted UniFrac (Lozupone and Knight, 2005), and using Weighted UniFrac (Lozupone and Knight, 2007). To determine which variables have a significant effect on variation in beta diversity, a permutational multivariate analysis of variance (PERMANOVA) test was performed using vegan::adonis2. Adonis2 applies a linear model to a given distance matrix using a sum of squares method for calculating R^2^ values to determine goodness of fit, and an F-test to determine significance (Oksanen et al., 2013). Tests for homogeneity of dispersion between groups of samples were performed using vegan::betadisper (Anderson and Walsh, 2013; The source for betadisper plot function can be found at: myplotbetadisp.r – Pastebin.com). The above-mentioned sequencing and analyses of stool samples were performed according to the same protocol as in our recent study in prairie voles (Donovan et al., 2020b).

### Correlation analyses

Correlations between the taxonomic features, the neurological and behavioral data, and the qPCR quantification were performed in the phyloseq R package using the Spearman correlation method (Salkind, 2012) which treats data in an ordinal fashion. To minimize false discovery, only taxonomic calls with non-zero hits in at least 10% of the total number of samples were kept for the correlations (Shi et al., 2016). Correlation significance was determined first by calculating non-zero confidence intervals at the 0.95 and 0.99 levels for the Spearman Rho values using the Bonett-Wight method for multiple test correction (Bonett and Wright, 2000). Significant correlation between the microbiota and the neurological, behavioral, or qPCR data was then declared if a percentage of taxonomic call rho values proportionate to the confidence interval alpha fell within the respective non-zero confidence interval (Shi et al., 2016).

## Results

### *L. reuteri* treatment effects on water intake and body weight

As *L. reuteri* MM4-1A was administered *via* drinking water, we assessed effects of *L. reuteri* administration on both daily water intake and on body weight (Supplementary Table S1). There were no significant treatment effect, sex difference, or sex-by-treatment interaction in body weight changes. There were neither treatment effect nor sex difference on average water intake. However, there was a sex-by-treatment interaction with average water intake; *post hoc* analyses indicated that male control subjects drank more water across the study compared to all other groups [*F*_(1,28)_ = 10.51, *p* < 0.01].

### Sex differences were found in anxiety-like behavior in both elevated plus maze and open field tests

**Figure 1:**
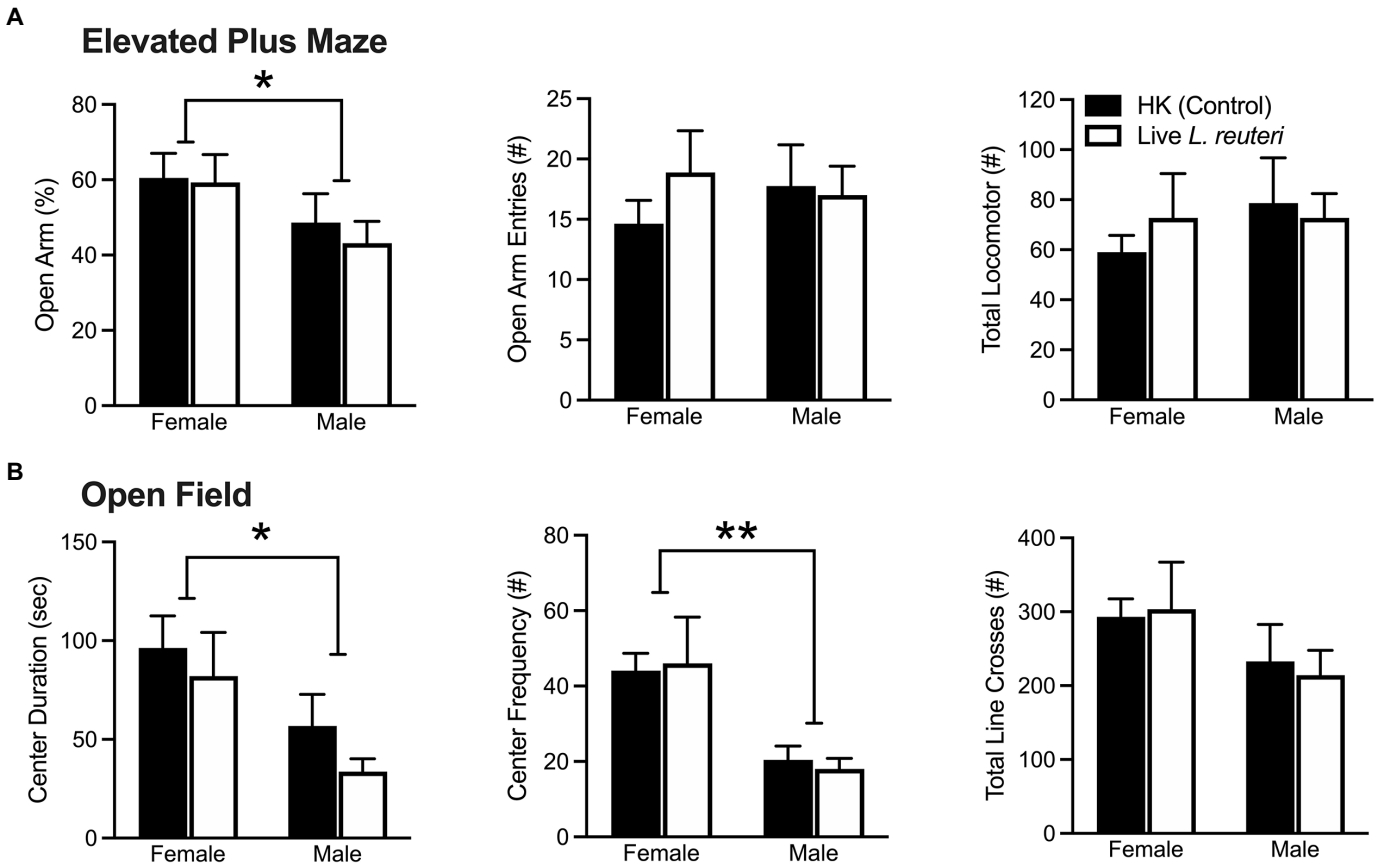
Females display significantly lower anxiety-like behaviors in both the EPM and OF tests. Females spent a higher percentage of time in the open arms of the EPM, but there were no differences in open arm entries nor total locomotor activity **(A)**. Females spent more time in and entered the center of the OF more often than males, but there were no differences in total locomotor activity **(B)**. EPM, elevated plus maze; OF, open field. Bars indicate mean ± SEM. * represents *p* < 0.05, ** represents *p* < 0.01.

Our data indicated overall sex differences in anxiety-like behavior in both the EPM and OF tests. In the EPM test (Figure 1A), female prairie voles had a higher percentage of time spent in the open arms of the EPM [*F*_(1,28)_ = 4.12, *p* < 0.05]. There were no sex differences in the frequency of open arm entries [*F*_(1,28)_ = 0.05, *p* = 0.83] or in overall locomotor activity [*F*_(1,28)_ = 0.49, *p* = 0.49]. No significant treatment effects nor sex-by-treatment interactions were found in the EPM test. In the OF test (Figure 1B), females spent more time in the center of the apparatus [*F*_(1,28)_ = 7.39, *p* < 0.05] and entered the center more frequently [*F*_(1,28)_ = 13.72, *p* < 0.01] in comparison to males. There were no sex differences in the number of total line crosses [*F*_(1,28)_ = 2.70, *p* = 0.11]. No other significant effects of treatment nor sex-by-treatment effects were found in the OF test.

When converting these data into z-scores to compare across behavioral assays, we replicated our effect, where females had a significantly lower average anxiety composite z-score in comparison to males [*F*_(1,28)_ = 7.42 *p* < 0.01]. Taken together, these data indicate that, compared to males, females consistently displayed lower levels of anxiety-like behavior.

### Social behavior differed between the *L. reuteri* treatment groups in a sex-dependent manner

**Figure 2:**
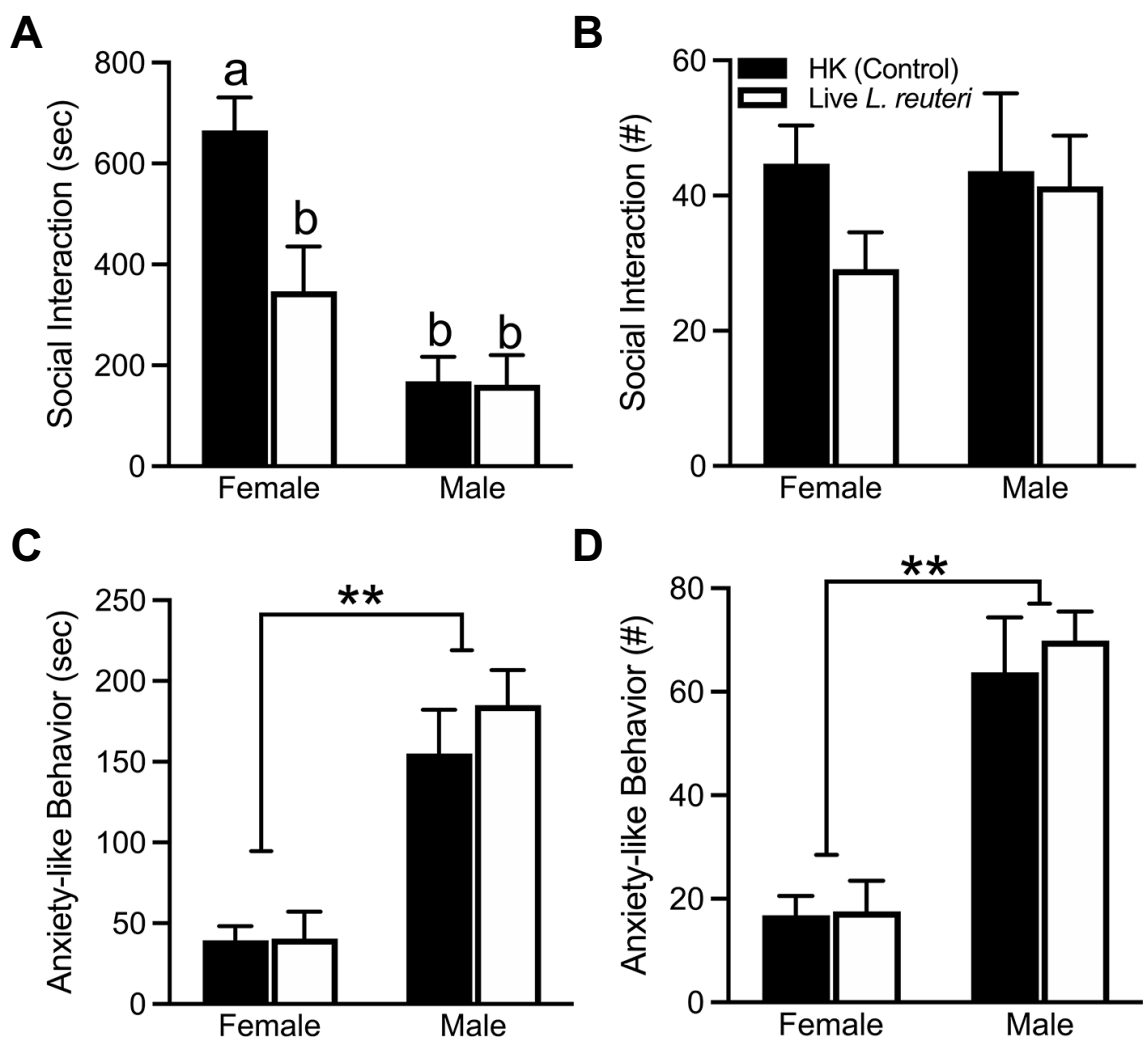
Live *L. reuteri* intake alters behaviors in the SA test. Females treated with live *L. reuteri* spent significantly less time interacting with an unfamiliar, same-sex conspecific in comparison to control females **(A)**. There were no differences in frequency of social interaction across treatment and sex **(B)**. Females displayed lower duration **(C)** and frequency **(D)** of anxiety-related behaviors (i.e., self-grooming, rearing) compared to males. SA, social affiliation. Bars indicate mean ± SEM. Bars with different letters differ significantly from each other. ** represents *p* < 0.01.

There were no differences in the total amount of time spent in either cage across treatment groups (Supplementary Table S2), as measured by the Social Affiliation test (SA). However, our data show a sex-by-treatment interaction in the duration the subject spent interacting with the conspecific [*F*_(1,26)_ = 5.68, *p* < 0.05], where live-treated females spent significantly less time interacting in comparison to HK-treated females. This phenomenon was not found in male voles (Figure 2A). There were no sex differences [*F*_(1,26)_ = 0.46, *p* = 0.50], treatment differences [*F*_(1,26)_ = 1.17, *p* = 0.29], nor sex-by-treatment interactions [*F*_(1,26)_ = 0.65, *p* = 0.43] in the total frequency of social interactions with the conspecific (Figure 2B). An overall sex difference was found in anxiety-like behavior (i.e., autogrooming and rearing), with females displaying both lower duration [*F*_(1,26)_ = 39.70, *p* < 0.01] and frequency [*F*_(1,26)_ = 47.11, *p* < 0.01] of anxiety-like behavior in comparison to male voles (Figures 2C,D). Additional behaviors quantified can be found in Supplementary Table S2.

### CRF/V1aR marker expression differed in selected brain areas between the live- and HK-*L. reuteri* treated groups

**Figure 3:**
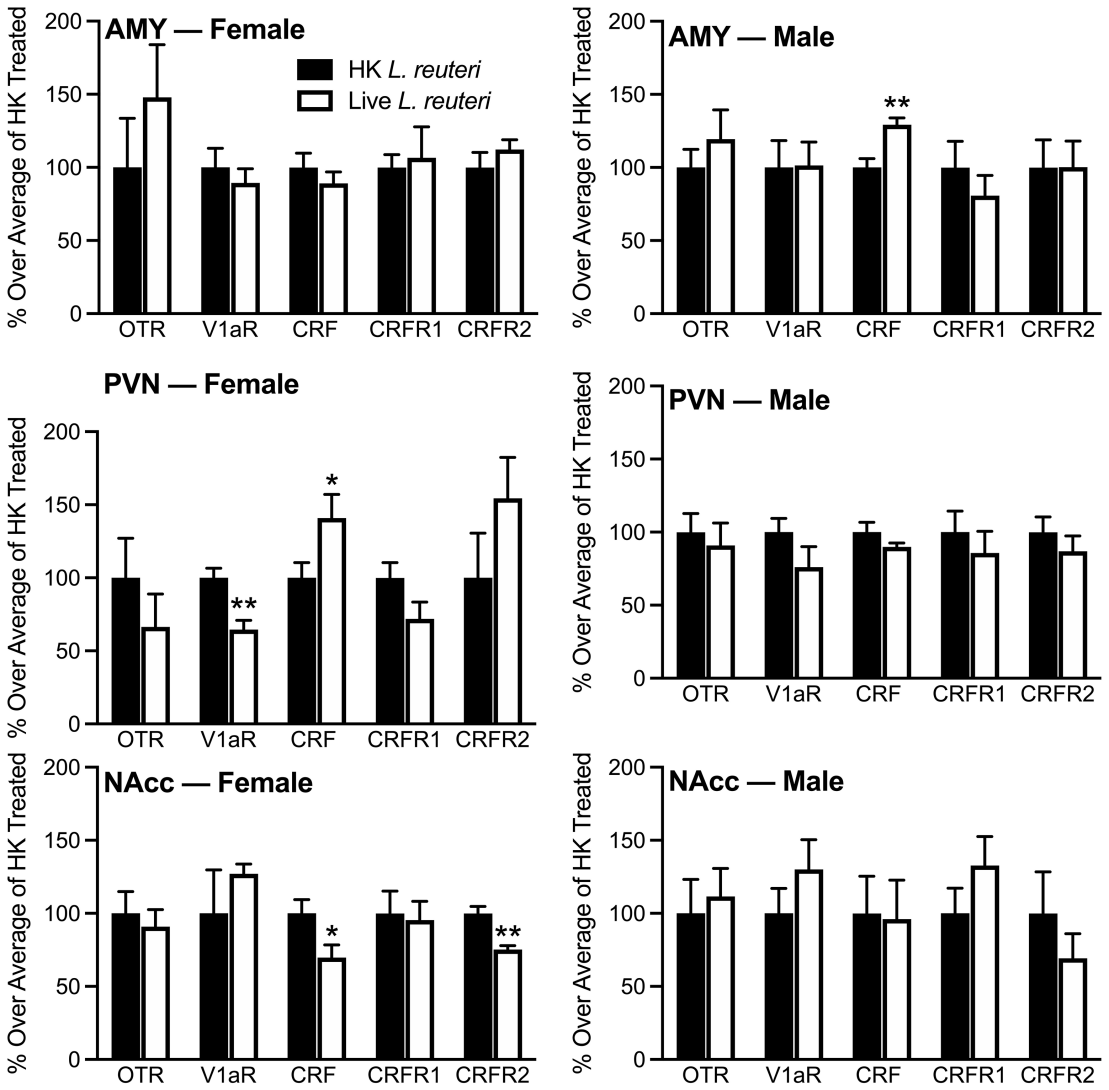
Live *L. reuteri* intake associated with altered neurochemical expression in a brain region-specific manner. Males that were treated with live *L. reuteri* had significantly increased CRF in the AMY compared to controls. Females treated with live *L. reuteri* had significantly increased CRF and decreased V1aR in the PVN. Live-treated females also had lower levels of CRF and CRFR2 compared to control females. AMY, amygdala; PVN, paraventricular nucleus of the hypothalamus; NAcc, nucleus accumbens; CRF, corticotropin releasing factor; V1aR, vasopressin 1a receptor; CRFR2, corticotropin releasing hormone receptor 2. Data are plotted as percent average over controls. Bars indicate mean ± SEM. * represent *p* < 0.05, ** represent *p* < 0.01.

A variety of neurochemical markers in selected brain areas known to be involved in anxiety-like and social behavior were assayed by Western blot and quantified (Figure 3). Our data show that live-treated female voles had significantly less expression of CRF [*F*_(1,13)_ = 5.55, *p* < 0.05] and CRFR2 [*F*_(1,14)_ = 20.46, *p* < 0.01] in the NAcc and V1aR in the PVN [*F*_(1,14)_ = 15.13, *p* < 0.01], but higher CRF expression in the PVN [*F*_(1,14)_ = 4.56, *p* < 0.05], compared to HK-treated females. In males, live-treated voles had greater CRF expression in the AMY in comparison to HK-treated ones [*F*_(1,13)_ = 15.38, *p* < 0.01]. No other significant treatment differences were found in measured neurochemical markers. Values for each neurochemical marker Western band density relative to the control GAPDH band density are shown in Supplementary Table S3 (females) and Supplementary Table S4 (males).

### Sex differences were found in the baseline microbiota compositions

**Figure 4:**
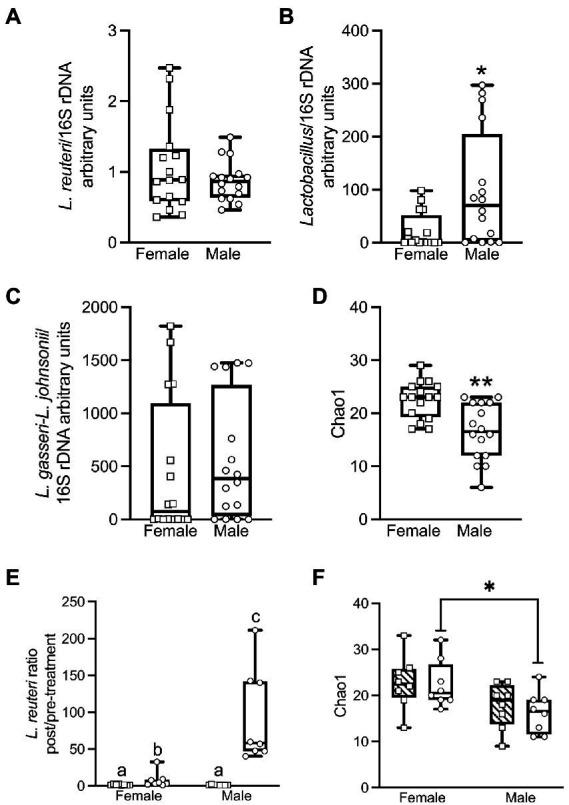
qPCR analysis shows that female and male voles have significant differences in microbiome composition prior to *L. reuteri* treatment. Females have more *L. reuteri* than males based on *L. reuteri* DNA relative to the 16S rRNA gene (ΔΔCt method), but this failed to reach significance (Mann–Whitney *U* = 121; *p* = 0.81; *r* = 0.04) **(A)**. Males have significantly more *Lactobacillus* than females (*Lactobacillus*/16S rRNA gene) (*U* = 68; *p* = 0.023; *r* = 0.4) **(B)**. There is no significant difference between males and females in *L. gasseri–L. johnsonii*/16S rRNA gene (*U* = 106; *p* = 0.42; *r* = 0.14) **(C)**. Prior to treatment, females have significantly greater bacterial family richness than males by the Chao1 index for abundance (*U* = 40.5; *p* < 0.001; *r* = 0.58) **(D)**. After treatment with *L. reuteri*, qPCR shows that the pre- to post-treatment fold change in *L. reuteri* is significantly greater in males than in females (*U* = 0; *p* < 0.001; *r* = 0.83). The difference is not significant between males and females treated with heat-killed *L. reuteri* (*U* = 28; *p* = 0.72; *r* = 0.09) **(E)**. Post live-treated females have significantly greater bacterial family richness than males by the Chao1 index for rare taxa (*U* = 10.5; *p* = 0.02; *r* = 0.56), but the post-treatment sex differences are not significant for heat-killed treated animals (*U* = 15.5; *p* = 0.09; *r* = 0.42) **(F)**. (See Supplementary Figure S4 for all qPCR and alpha-diversity statistical tests).

We assessed pre-treatment stool samples for overall sex differences in baseline microbiota composition. qPCR analysis showed that untreated females vs. males had no significant difference in *L. reuteri* relative to the 16S rRNA gene V3-V4 amplicon (ΔΔCt) (Mann–Whitney test for two independent samples *U* = 121, *p* = 0.81, *r* = 0.04) (Figure 4A). (See Supplementary Table S5 for qPCR relative quantifications, and Supplementary Table S7 for qPCR pairwise statistical tests.) In contrast, untreated males had a significant 4.4-fold higher abundance of *Lactobacillus* than females (*U* = 68, *p* = 0.023, *r* = 0.40). (Figure 4B). Prior to treatment there was no significant difference (Supplementary Table S7) between males and females (Figure 4C) in the quantity of the abundant *L. gasseri/L. johnsonii* group of strains, members of which have previously been isolated from prairie vole intestine (Assefa et al., 2015). There were no significant differences in any of these *Lactobacillaceae* taxa between the animals that were subsequently treated with live *L. reuteri* vs. those subsequently treated with HK *L. reuteri* (Supplementary Table S7). The alpha diversity of the female stool 16S rRNA gene microbiota measured by V3-V4 amplicon sequencing prior to *L. reuteri* treatment showed significantly greater richness than that of males according to the Chao1 estimator for rare taxa (Chao, 1984) at the family level (MW *U* = 40.5, *p* < 0.001, *r* = 0.58) (Figure 4D), and at the species level (*U* = 58, *p* = 0.007, *r* = 0.46). The pre-treatment alpha diversity was also greater for females at the family level measured by the Shannon ENS diversity metric (Shannon and Weaver, 1949), which weights taxa richness over evenness (*U* = 66, *p* = 0.019, *r* = 0.41). There were no significant differences in the Inverse Simpson’s diversity metric, which weights taxa evenness over richness (Simpson, 1949; Supplementary Table S5, alpha-diversity values; Supplementary Table S7, pairwise statistical tests).

### *L.reuteri* treatment altered microbiome composition in a sex-dependent manner

We examined the impact of *L. reuteri* administration on microbiome composition in male and female prairie voles. Due to the high level of individual variation between animals, the fold change pre- to post-treatment was determined for each individual animal and the average fold change for the group was calculated from these individual fold changes. Inoculation with live *L. reuteri* for 28 days produced a mean 93.2-fold increase in males (Figure 4E) in *L. reuteri* DNA detected by qPCR (relative to 16S rRNA gene V3-V4 amplicon) and a 7.9-fold increase in females. The difference in fold change between the sexes was highly significant (Mann–Whitney *U* = 0, *p* < 0.001, *r* = 0.83). We also found that the relative quantities of *L. reuteri* in post- vs. pre- live-treated animals were significantly higher for both males (Wilcoxon matched-pairs signed-rank test *W* = 36, *p* = 0.008) and females (*W* = 36, *p* = 0.008). Treatment with HK *L. reuteri* produced a very small 1.3-fold increase in *L. reuteri* in both males and females with no difference between the sexes (*U* = 28, *p* = 0.72, *r* = 0.09). The difference between pre- vs. post-HK treatment groups was not significant for either sex (Supplementary Table S7). Differences in *L. reuteri* in the post-live vs. post-HK treatment groups were significant for both males (*U* = 0, *p* < 0.001, *r* = 0.83) and females (*U* = 9, *p* = 0.015, *r* = 0.59), but the differences for pre-treatment groups were not significant for either sex (Supplementary Table S7). The only significant difference in *Lactobacillus* between the treatment groups was in the post live- vs. post HK-treated groups in females (*U* = 7, *p* = 0.007, *r* = 0.64). There were no significant differences in the *L. gasseri–L. johnsonii* species group between the treatment groups (Supplementary Table S7).

Measured by 16S rRNA gene sequencing, after live *L. reuteri* treatment, females had significantly greater family-level richness than males by Chao1 (*U* = 10.5, *p* = 0.021, *r* = 0.56; Figure 4F), and greater species-level richness by the same metric (*U* = 12, *p* = 0.034, *r* = 0.51; Chao, 1984). In contrast, the difference between Chao1 family-level richness between females and males was not significant after treatment with HK *L. reuteri* (*U* = 15.5, *p* = 0.087, *r* = 0.42; Figure 4F). There were no significant differences in the male vs. female post-live-treated or post-HK-treated samples by the Shannon ENS estimator (Shannon and Weaver, 1949) or the Inverse Simpson’s diversity metric (Simpson, 1949). There were no significant alpha diversity differences by 16S rRNA gene analysis between same-sex groups treated with live- vs. HK *L. reuteri* by Chao1, Shannon ENS, or Inverse Simpson’s. There were also no significant differences in pre-treatment vs. post-treatment samples by these 3 metrics. In comparisons of WGS data (obtained only for females) between sample groups, there were no significant differences in alpha diversity by Chao1, Shannon ENS, or Inverse Simpson’s in the live- vs. HK-treated or pre- vs. post-treatment groups. This is in agreement with the 16S rRNA gene data alpha diversity for females (Statistics shown in Supplementary Table S7. See Supplementary Table S5 for alpha diversity values).

There were significant sex differences in the composition of the microbiomes determined by comparing beta diversity metrics of the DESeq2-normalized 16S rRNA gene data in a PERMANOVA using adonis2. (Results are shown in Table 1, with significant differences marked with (*). Adonis2 formulas are given in Supplementary Table S8). When all samples were considered, significant sex differences were found at the species level by Bray–Curtis dissimilarity (Bray and Curtis, 1957), Unweighted Unique fraction metric (UniFrac) (Lozupone and Knight, 2005), and Weighted UniFrac (Lozupone et al., 2007) and at the family level by Bray–Curtis dissimilarity and UniFrac (*p* values shown in Table 1). The sex differences were significant by UniFrac at both the species and family level in pre- and post-treatment samples, and by Bray-Curtis in post-treatment samples at the species level (Table 1). When live-treated and HK-treated animals were considered separately, the sex differences were also significant for both live and HK-treated animals by Bray-Curtis and UniFrac at the species level. At the family level, sex differences were significant by Bray-Curtis and UniFrac for HK-treated animals and by UniFrac for live-treated animals (Table 1). Significant sex differences by UniFrac, but not Weighted UniFrac suggests the microbiota differences may be in less abundant lineages. There were no significant differences in beta diversity for pre- vs. post-treatment samples (Table 1). There were also significant beta diversity differences in probiotic treatment. In live- vs. HK treatment groups, when all 16S rRNA gene samples were considered, there were significant differences in beta diversity at the species level and at the family level by Bray-Curtis and Weighted UniFrac (Table 1). Among only the female samples, there were significant differences in the treatment groups by 16S rRNA gene analysis in the Bray–Curtis dissimilarity at both the species and family level and in UniFrac at the species level (Table 1). The only significant difference in beta diversity in the female WGS groups was between live- and HK-treated animals at the family level by the Bray–Curtis dissimilarity (*p* = 0.050). Additional differences in composition between live- vs. HK treatment groups were only for 16S rRNA gene analysis and only at the family level. Live- vs. HK-treated males differed significantly by Bray-Curtis and Weighted UniFrac (Table 1). The pre-treatment samples, but not the post-treatment samples differed by probiotic treatment at the family level in Bray–Curtis dissimilarity (Table 1).

**Table 1:** PERMANOVA of the effects of variables on 16S rDNA beta-diversity determined using adonis2.

		Stool samples	Bray-Curtis		Unweighted UniFrac		Weighted UniFrac	
	**Variables**		***R*** ^**2**^	***P***	***R*** ^**2**^	***P***	***R*** ^**2**^	***P***
Species-level differences	Sex	All	0.047	0.001*	0.059	0.001*^#^	0.037	0.006*^#^
	Probiotic treatment	All	0.031	0.009*^#^	0.016	0.111	0.032	0.010*^#^
	Pre- vs. Post- †	All	0.014	0.979	0.026	0.306	0.013	0.856
	Probiotic treatment	Female	0.054	0.040*	0.051	0.026*	0.058	0.063
	Pre- vs. Post-	Female	0.042	0.996	0.058	0.586	0.042	0.889
	Probiotic treatment	Male	0.049	0.083^#^	0.037	0.237	0.044	0.141^#^
	Pre- vs. Post-	Male	0.035	0.955	0.045	0.904	0.038	0.637
	Sex	Pre-	0.048	0.070	0.084	0.001*^#^	0.042	0.168
	Probiotic treatment	Pre-	0.05	0.064^#^	0.033	0.322	0.052	0.102^#^
	Sex	Post-	0.064	0.014*	0.076	0.001*^#^	0.051	0.100^#^
	Probiotic treatment	Post-	0.027	0.573	0.029	0.510	0.026	0.510
	Sex	HK	0.065	0.030*	0.080	0.004*^#^	0.055	0.082^#^
	Pre- vs. Post-	HK	0.007	0.999	0.022	0.749	0.008	0.964
	Sex	Live	0.107	0.004*	0.090	0.001*^#^	0.054	0.073
	Pre- vs. Post-	Live	0.024	0.753	0.031	0.378	0.021	0.759
Family-level differences	Sex	All	0.038	0.010*^#^	0.099	0.001*^#^	0.024	0.086^#^
	Probiotic treatment	All	0.046	0.002*^#^	0.014	0.297	0.057	0.005*^#^
	Pre- vs. Post- †	All	0.021	0.340	0.021	0.591	0.013	0.668
	Probiotic treatment	Female	0.068	0.044*	0.034	0.356	0.068	0.096
	Pre- vs. Post-	Female	0.032	0.949	0.054	0.567	0.025	0.932
	Probiotic treatment	Male	0.071	0.043*^#^	0.040	0.275	0.081	0.039*^#^
	Pre- vs. Post-	Male	0.045	0.513	0.035	0.925	0.023	0.820
	Sex	Pre-	0.04	0.213	0.117	0.001*^#^	0.025	0.389
	Probiotic treatment	Pre-	0.085	0.029*^#^	0.029	0.446	0.097	0.019*
	Sex	Post-	0.060	0.067^#^	0.102	0.009*^#^	0.034	0.311
	Probiotic treatment	Post-	0.026	0.480	0.016	0.819	0.036	0.291
	Sex	HK	0.067	0.048*^#^	0.109	0.001*	0.043	0.173^#^
	Pre- vs. Post-	HK	0.023	0.481	0.019	0.715	0.014	0.619
	Sex	Live	0.056	0.091	0.133	0.003*	0.046	0.183
	Pre- vs. Post-	Live	0.021	0.685	0.023	0.577	0.014	0.779

†Nest pre-probiotic treatment vs nested post-probiotic treatment, * Significant at *p* < 0.05. # Significant difference in dispersion (See Supplementary Figure S2).

In determinations of beta-diversity, if one group has very little variation among the samples that make up the group (a low level of dispersion) and the other group has very large variation (a high level of dispersion), this can have an impact on the significance of the beta-diversity metrics, although PERMANOVA is less sensitive to sample dispersion differences than other analysis methods (Anderson and Walsh, 2013). We tested for differences in dispersion between the sample groups using betadisper (Oksanen et al., 2013) and those with significant differences are marked in Table 1 with a # sign. For each of the significant differences in beta-diversity metrics shown in Table 1, a PCoA plot showing the sample dispersion is shown in Supplementary Figure S2. The differences in dispersion prior to *L. reuteri* treatment suggest large variations in microbiome composition between animals. To minimize these dispersion differences in our subsequent analyses of differences in specific taxa, we determined the log2 fold change (log2fc) on a ‘Vole-by-Vole’ (VBV) pairwise basis for each individual animal prior to comparison of the sample groups (see Methods).

### The relative abundances of taxa detected in the prairie vole stool microbiome differed between the 16S rRNA gene V3-V4 amplicon and WGS data sets

**Figure 5:**
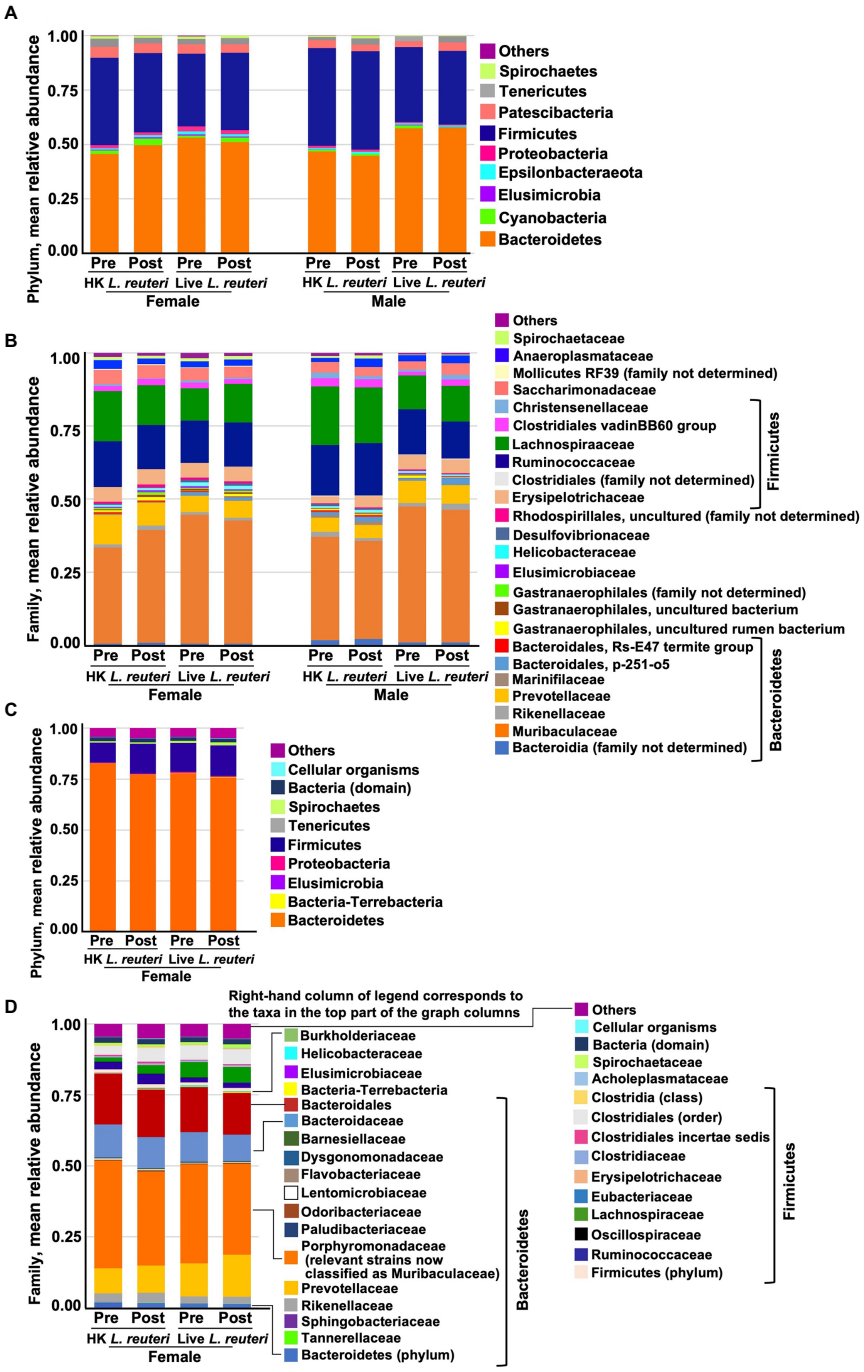
Mean relative abundance of bacterial taxa in stool measured by, 16S rRNA gene V3-V4 amplicon sequencing in the different groups at the phylum **(A)** and family **(B)** level, and by WGS at the phylum **(C)** and family **(D)** level. *Bacteroidetes* was the average most abundant phylum across the groups by 16S rRNA gene (51% overall average), orange bars in **(A)**. *Muribaculaceae* (formerly S24-7) was the most abundant family in all groups of animals by 16S rRNA gene analysis (40% overall average), orange bars in **(B)**. *Bacteroidetes* was the average most abundant phylum across the groups by WGS (78% overall average), orange bars in **(C)**. *Porphyromonadaceae* (in which the most abundant strains of *Muribaculaceae* were formerly classified) was the most abundant family in all groups by WGS (34% overall average), orange bars in **(D)**.

Measured by the 16S rRNA gene V3-V4 amplicon, the average most abundant phylum in vole stool was *Bacteroidetes* (50–58% of bacteria across the sample groups; Figure 5A), with most of these in the family *Muribaculaceae/*S24-7 (39–47% of bacteria) or the family *Prevotellaceae* (4–8%; Figure 5B). Next in abundance was the Firmicutes phylum (34–41%; Figure 5A) with most of this split between the *Ruminococcaceae* (12–17% of bacteria) and the *Lachnospiraceae* (11–18%; Figure 5B; Details shown in Supplementary Figures S3–S6). These data are similar to our previously-published 16S rRNA gene V3-V4 amplicon results (Donovan et al., 2020b). As in the 16S rRNA gene analysis, the most abundant phylum measured by WGS sequencing of female stool samples was *Bacteroidetes* (Figure 5C), but the percent *Bacteroidetes* across the female samples appears much greater by WGS (average 78–85%) than by 16S rRNA gene V3-V4, despite the fact that these analyses were conducted on the same DNA samples. The most abundant strain was classified by QIIME2 in 16S rRNA gene analysis as a *Muribaculaceae* (S24-7) uncultured bacterium (36–42%, Supplementary Figures S3–S6), whereas it was classified by the Metagenome-ATLAS pipeline in the WGS analysis as *Parabacteroides* YL27 (22–26% of bacteria across the sample groups, Supplementary Figures S3–S6). *Parabacteroides* YL27 was formerly classified in the *Porphyromonadaceae* family rather than the *Muribaculaceae* family (Ormerod et al., 2016), and this is responsible for *Porphyromonadaceae* rather than *Muribaculaceae* appearing as the most abundant family in the WGS analysis (Figure 5D and Supplementary Figures S3–S6). *Firmicutes* was the second most abundant phylum by WGS (12–18% of bacteria), with most of this split between the *Ruminococcaceae* (2–4% of bacteria) and the *Lachnospiraceae* (2–6%). Since the 16S V3-V4 analysis and the WGS analysis were done on the same DNA samples, the results suggest bias is introduced by one or both of these methods. The more likely possibility is that the V3-V4 amplicon primers have a bias against detection of one or more *Bacteroidetes* strains in our samples. The MAGs extracted from our WGS vole data have poor recovery of 16S rRNA genes (Donovan et al., 2020a), so it has not yet been possible to determine if this is the case. A second, less likely, possibility is that there is bias toward Bacteroidetes in the WGS workflow, for example in the mechanical shearing and/or fragment size selection steps (Poptsova et al., 2014). Despite these differences in taxa abundance, there were no significant differences in alpha diversity between 16S rRNA gene and WGS datasets by Shannon ENS or Inv Simpson’s for either female sample group (Alpha diversity values shown in Supplementary Table S5. Mann–Whitney test statistics shown in Supplementary Table S7). However, a significantly larger number of rare families were detected in the WGS data for both treatment groups by the Chao1 estimator for rare taxa (*U* = 0, *p* = 0.0002, *r* = 0.83).

### *L.reuteri* treatment altered the prevalence of individual bacterial taxa detected by 16S rRNA gene analysis

**Figure 6:**
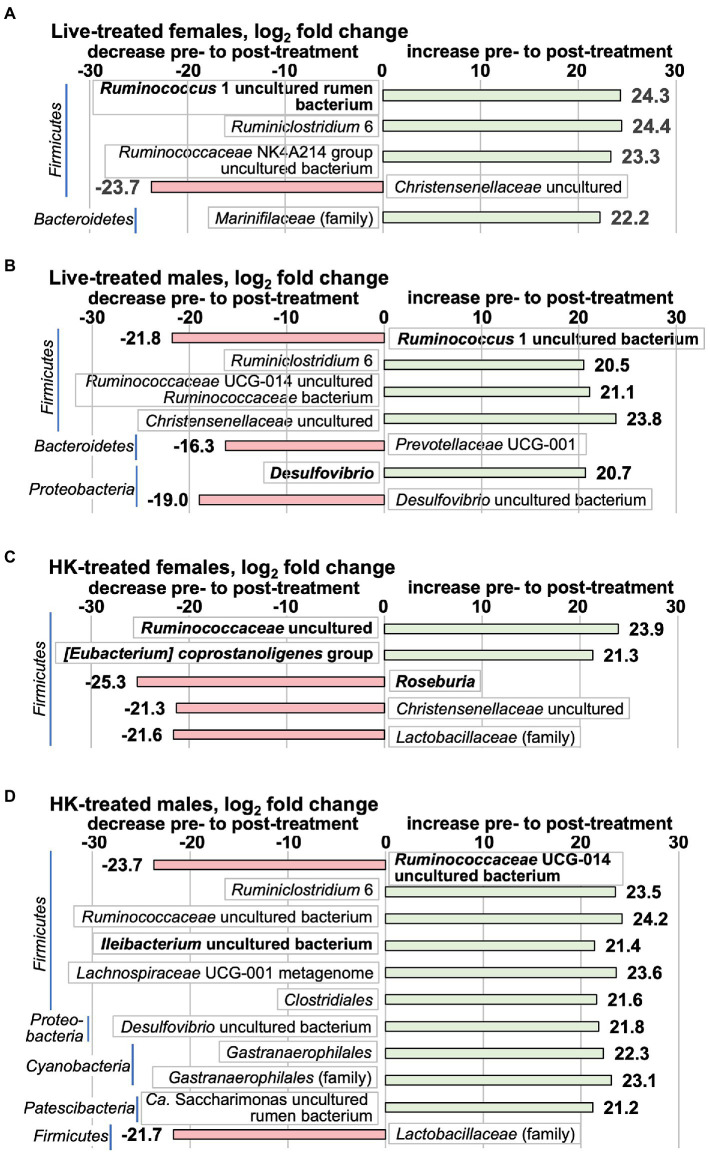
Taxa that change by the whole-group method pre- to post-*L. reuteri* treatment measured by 16S rRNA gene V3-V4 amplicon. Positive log_2_fc numbers indicate an increase pre- to post-treatment and are shown with green bars, whereas negative numbers indicate a decrease, and are shown with red bars. Changes pre- to post-treatment are **(A)** live-treated females; **(B)** live-treated males; **(C)** HK-treated females; **(D)** HK-treated males. Graphs show all taxa that change with *p* adj < 0.1. Taxa shown in bold are in the highest relative abundance quartile in the pretreatment group. All differential abundance values and *p* values are shown in Supplementary Tables S9–S12.

We used two methods to analyze the 16S rRNA gene V3-V4 amplicon data for changes pre- to post-treatment in bacterial taxa. First, the differences in DESeq2-normalized abundance of taxa post- relative to pre-treatment were compared for whole groups [Figure 6 shows significant results (*p* < 0.1 adjusted for multiple comparisons) Supplementary Tables S9–S12 show full results]. When analyzed by this method, only 8 species with significant pre- to post-treatment changes were in the highest abundance quartile prior to treatment (taxa shown in bold in Figure 6). The only change in live-treated females in a top abundance quartile bacterium was a *Ruminococcus* 1 uncultured rumen bacterium that increased post-treatment. In live-treated males, a different *Ruminococcus* 1 species decreased, and a *Desulfovibrio* genus bacterium increased. In HK-treated females, another species of the *Ruminococcaceae* family and an *[Eubacterium] coprostanoligenes* group species increased, whereas a *Roseburia* species decreased. In HK-treated males an *Ileibacterium* uncultured bacterium increased and a *Ruminococcaceae* UCG-014 uncultured bacterium decreased. At the family level, by the whole group method, only 3 bacterial families had significant changes and all of these were in lower-abundance lineages (Figure 6). Surprisingly, the *Lactobacillaceae* family decreased in both HK-treated females and males (Figure 6) even though no significant changes in *Lactobacillus* were detected in HK-treated animals by qPCR (Supplementary Table S7).

**Figure 7:**
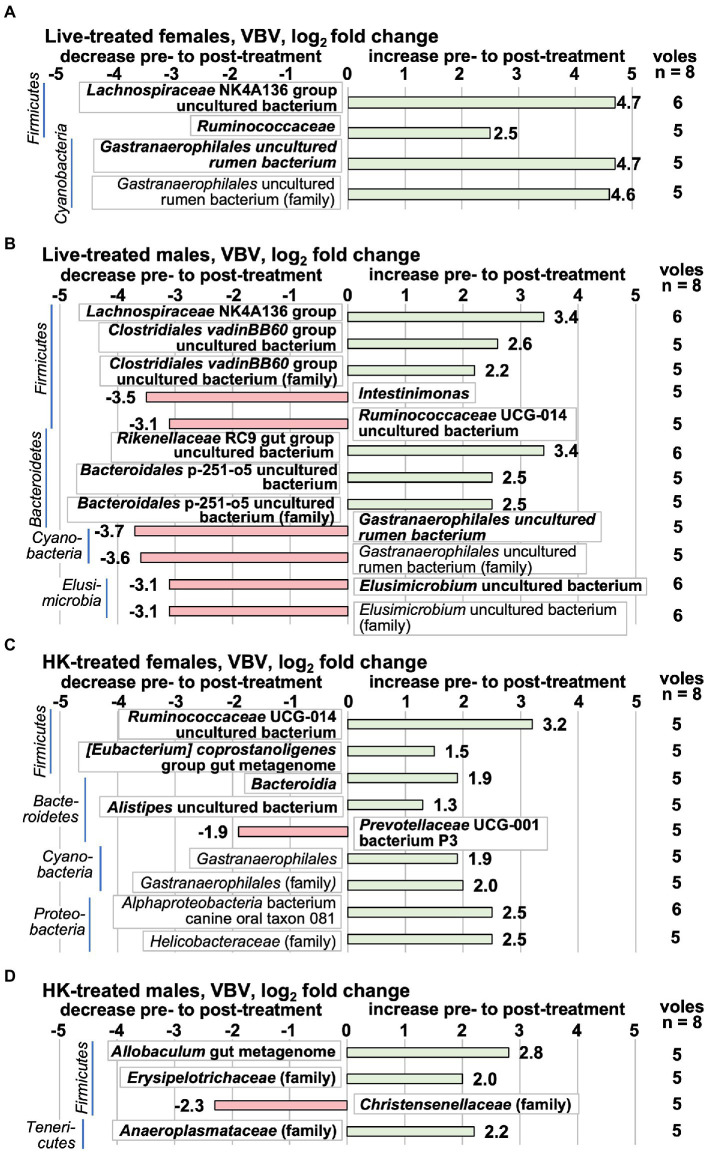
Taxa that change pre- to post-*L. reuteri* treatment tested ‘vole-by-vole’ (VBV), measured by the 16S rRNA gene V3-V4 amplicon. Positive log_2_fc numbers indicate an increase pre- to post-treatment and are shown with green bars, whereas negative numbers indicate a decrease, and are shown with red bars. Changes are shown pre- to post-treatment in **(A)** live-treated females; **(B)** live-treated males; **(C)** HK-treated females; **(D)** HK-treated males. Taxa are shown in which a supermajority of voles each change individually by log_2_fc ≥ |0.693| in the same direction and the average log_2_fc for all 8 animals in the treatment group is ≥|1.25|. The value shown next to the bar is the average log_2_fc for all 8 animals in the treatment group. The column on the right shows the number of animals in the supermajority. (There were no taxa for which the supermajority consisted of 7 or 8 animals.) Taxa shown in bold are in the highest relative abundance quartile in the pretreatment group. All differential abundance values and *p* values are shown in Supplementary Tables S13, S14.

In this study and our previous work (Donovan et al., 2020b), we observed a great deal of variation between individual animals in taxa abundance in pre-treatment samples. In order to minimize the effect of individual differences between animals, and to focus on changes that coincided with *L. reuteri* treatment, differences in microbiota were also analyzed by determining the pre- to post- treatment log2fc on a ‘vole-by-vole’ (VBV) pairwise basis (see Methods). Taxa that had an individual log2fc of |0.693| in the same direction in at least 5 out of 8 animals and had an average log2fc of |1.25| for all 8 animals are shown in Figure 7. This method selects for taxa that change more evenly across the members of the group though the log2fc is not as dramatic as those detected by DESeq2 comparison of whole groups. The majority of the taxa detected by the VBV method are in the top abundance quartile (Figure 7), and there is little overlap between the taxa detected by this method and by the whole-group comparison method (Figure 6). (The log_2_fc in each animal for each species is shown in Supplementary Table S13 and for each family in Supplementary Table S14). The most striking alterations to the 16S rRNA gene-detected microbiota in a majority of live-treated females at the species level were increases in an uncultured bacterium from the *Lachnospiraceae* NK4A136 group, a species from the *Ruminococcaceae* family, and an uncultured rumen bacterium from the *Gastranaerophilales* (Figure 7). Live-treated males had a decrease in the same *Gastranaerophilales* uncultured rumen bacterium that increased in live-treated females. In HK-treated females, a different *Gastranaerophilales* species increased (Figure 7). This same *Gastranaerophilales* species increased in HK-treated males by the whole-group method (Figure 6). A *Ruminococcaceae* UCG-014 uncultured bacterium that increased in HK-treated females decreased in live-treated males (Figure 7), and by the whole-group method decreased in HK-treated males (Figure 6). Also increasing in HK-treated females were an undefined bacterium of the *Bacteroidia* class, an *[Eubacterium] coprostanoligenes* gut metagenome bacterium, an uncultured *Alistipes* bacterium and a canine oral taxon 081 *Alphaproteobacterium*. The only species that decreased in HK-treated females was *Prevotellaceae* UCG-001 bacterium species P3. An increase in an *Allobaculum* gut metagenome species was the only species-level change observed in HK-treated males. There was little overlap between the changes observed in the live-treated males and changes in the other treatment groups. The *Lachnospiraceae* NK4A136 group species that increased in live-treated males is different from the one that increased in live-treated females. Also increasing in live-treated males were species from the *Rikenellaceae* RC9 gut group, the *Clostridiales* vadinBB60 group (uncultured bacterium), and the *Bacteroidales* p-251-o5. Decreasing in the live-treated males were species from the *Elusimicrobium* and the *Intestinimonas* genera, in addition to the aforementioned *Gastranaerophilales* uncultured rumen bacterium. All of the family-level differences in live-treated animals were in groups also detected at the species level. In HK-treated females there were increases in a *Bacteroidia* family, the *Helicobacteriaceae*, and a family of *Gastranaerophilalaes*. In HK-treated males, the *Anaeroplasmataceae* and *Erysipelotrichaceae* families increased, whereas the *Christensenellaceae* family decreased.

**Figure 8:**
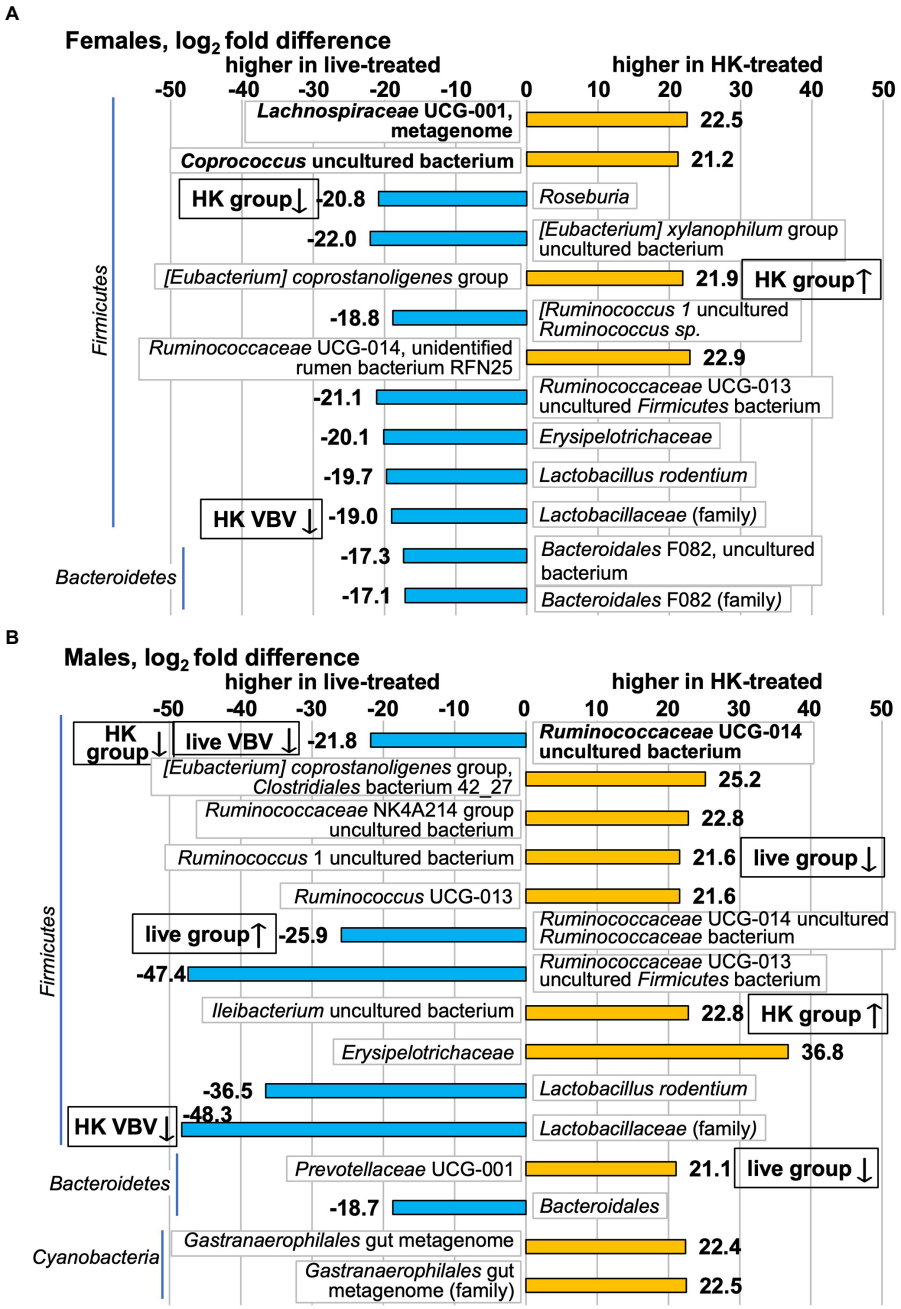
Taxa that differ in post-treatment relative abundance in HK-treated vs. live-treated voles measured by 16S rRNA gene V3-V4 amplicon. Positive log_2_fc numbers indicate higher relative abundance in HK-treated voles and are shown with orange bars, whereas negative numbers indicate higher relative abundance in live-treated voles and are shown with blue bars. Differences shown in **(A)** Females; **(B)** Males. Graphs show all taxa that differ with *p* adj < 0.1. Taxa shown in bold are in the highest relative abundance quartile in the pretreatment group. All differential abundance values and *p* values are shown in Supplementary Tables S15, S16. Boxes with up or down arrows next to graph bars indicate an increase or decrease was observed in HK- or live-treated groups in either Figure 6 (group) or Figure 7 (VBV).

These pre- to post-*L. reuteri* treatment changes produced post-treatment differences in some of these taxa between live- vs. HK-treated groups. Of 11 species that were differentially abundant between live-treated and HK-treated females (Figure 8; full results in Supplementary Table S15), a change was detected pre- to post-treatment for 2 of them by the whole-group method (Figure 6). The *[Eubacterium] coprostanoligenes* group species that increased in HK-treated females (Figure 6) is higher in HK-treated females than in live-treated (Figure 8). The *Roseburia* genus decreased in HK-treated females (Figure 6) and is higher in live-treated females (Figure 8). In males, 12 species were differentially abundant between the two post-treatment groups (Figure 8; full results in Supplementary Table S16) and 5 of those changed pre- to post-treatment (Figures 6, 7). Higher in live-treated males than in HK (Figure 8) due to changes pre- to post-treatment (Figure 6) were *Ruminococcaceae* UCG-014 uncultured bacterium and *Ruminococcaceae* UCG-014 uncultured *Ruminococcaceae* bacterium. Higher in HK-treated males than in live-treated (Figure 8) due to a change pre-to-post-treatment (Figure 6) were *Ileibacterium* uncultured bacterium, *Ruminococcus* 1 uncultured bacterium, and *Prevotellaceae* UCG-001. At the family level, higher levels of the *Lactobacillaceae* family were detected in live-treated males and females than in HK-treated males and females (Figure 8) and a decrease after HK-treatment was detected in both (Figure 6).

### WGS analysis detected changes in response to *L. reuteri* treatment that were not detected by 16S rRNA gene analysis, particularly in the *Actinobacteria*

**Figure 9:**
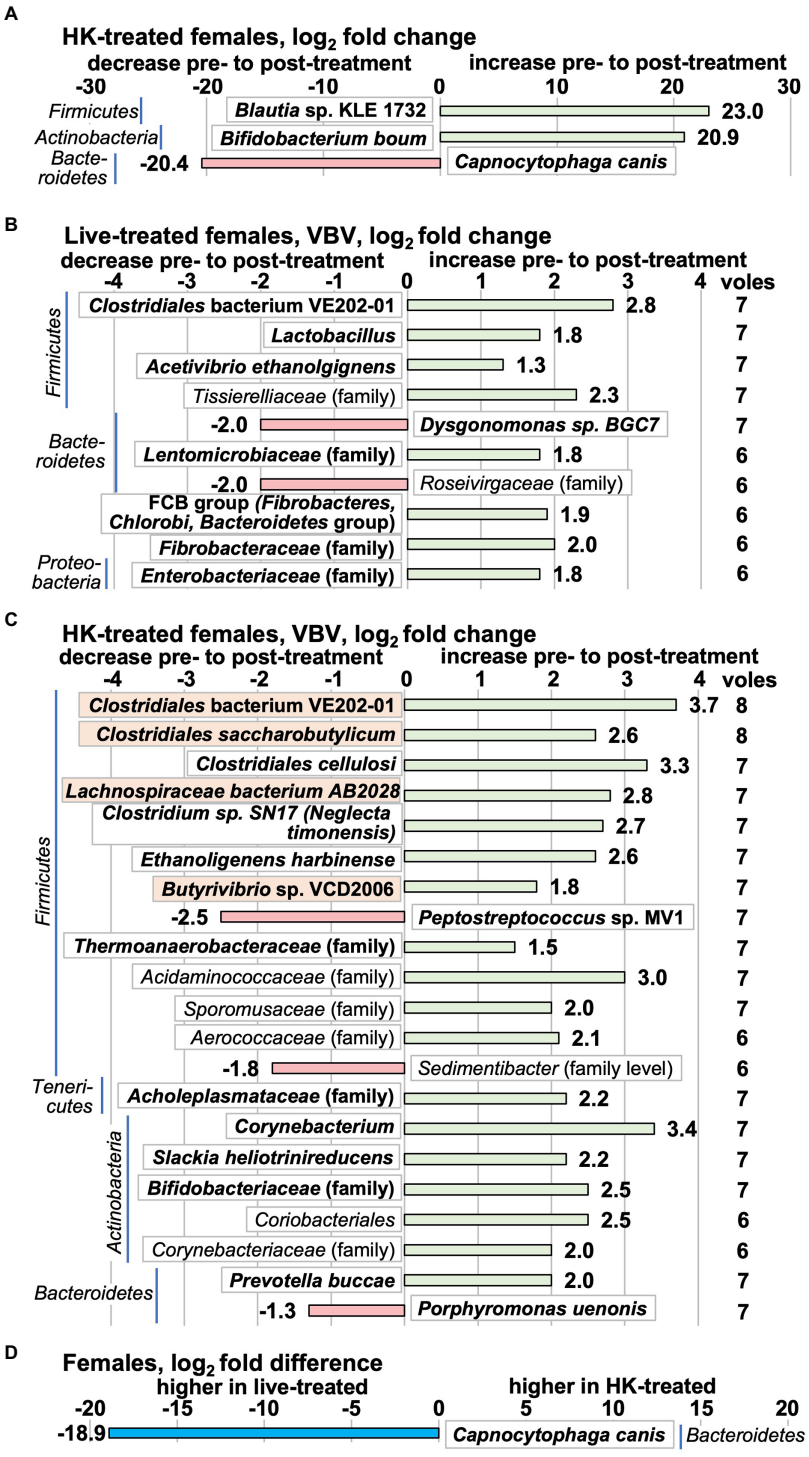
Microbiota differences in females measure by whole-genome shotgun (WGS) sequencing. For **(A–C)**, positive log_2_fc numbers indicate an increase pre- to post-treatment, whereas negative numbers indicate a decrease. **(A)** Taxa that change by the whole-group method pre- to post-treatment in HK-treated females. (No taxa that changed in live-treated females met the cut-off criteria of *p* adj < 0.1.) All differential abundance values and *p* values are shown in Supplementary Tables S17, S18. **(B)** Taxa that change by the ‘vole-by-vole’ (VBV) method pre- to post-live *L. reuteri* treatment. All taxa are shown in which 8–7 voles change by log_2_fc = |0.693| in the same direction. Families that change in 6–7 out of 8 animals are shown here if the average log_2_fc ≥ |1.8| for all 8 animals in the group. The column on the right shows the number of animals that met these criteria. The value presented in the bar graph is the average (Continued)FIGURE 9 (Continued)log_2_fc from pretreatment to posttreatment for all 8 animals in the treatment group (cut-off |1.8|). Supplementary Table S19 shows all taxa that change in the same direction by a log_2_fc of ≥ |0.693| in 5 out of 8 HK-treated animals and also have an average log_2_fc of ≥|1.25| for all 8 animals in the group. **(C)** Taxa that change by the VBV method pre- to post-HK *L. reuteri* treatment. The criteria for presentation here are the same as those described for **(B)**. Supplementary Table S20 shows full results for **(C)**. **(D)** The only taxon that differs in post-treatment relative abundance in HK-treated vs. live-treated female voles measured by WGS is *Capnocytophaga canis*, which is higher in live-treated animals due to the decrease in HK-treated animals shown in **(A)**. Species known to be or likely to be capable of butyrate production are shown in orange. Taxa shown in bold are in the highest relative abundance quartile in the pretreatment group.

As we saw behavioral changes in female, but not male voles, we analyzed the female samples *via* WGS sequencing to supplement the 16S rRNA gene analyses. Analysis of the WGS data by the whole-group method detects only 3 taxa with significant changes pre- to post-treatment, all in HK-treated females (Figure 9A; Supplementary Tables S17, S18). *Bifidobacterium boum* and the likely gallic acid metabolizer *Blautia* sp. *KLE 1732* (Esteban-Torres et al., 2018) increased while the pathogenic species *Capnocytophaga canis* decreased (Donner et al., 2020). We also performed VBV analysis to determine pre- to post-treatment fold changes per animal in the WGS data. In live-treated females 71 species increased by a log_2_fc of ≥0.693 in 5/8 animals and had an average increase across the 8 animals of log_2_fc > 1.25, whereas 28 species decreased (Figure 9B; Supplementary Table S19). In HK-treated females 101 species increased, and 27 decreased (Figure 9C; Supplementary Table S20). Four to 10% percent of species changed in the same direction in the two treatment groups and 0–4% changed in the opposite direction (See Supplementary Tables S19, S20 for full results). All species are shown in Figures 9B,C in which 8–7 voles change by log2fc = |0.693| in the same direction. Families that change in the same dirction in 6–8 animals are shown if the average log2fc ≥ |1.8| for all 8 animals in the group. All of the species that met the criteria to be shown in Figures 9B,C are in the highest abundance quartile. The WGS VBV comparison method detected an increase in *Lactobacillus* in live-treated females (Figure 9B). Other notable changes in live-treated animals (Figure 9B; Supplementary Table S19) were increases in *Clostridiales* bacterium VE202-01, the ethanol-producing species *Acetivibrio ethanolgignens* (Robinson and Ritchie, 1981), and the *Fibrobacteres, Chlorobi*, and *Bacteroidetes* (FCB) group. Decreasing in live-treated females was the xylan-degrading species *Dysgonomonas* sp. BGC7 (Bridges et al., 2021). At the family level in live-treated females, the WGS VBV had an increase in *Fibrobacteraceae* and also an increase in the lower-abundance family *Tissierelliaceae* (Figure 9B). The abundant proteobacterial family *Enterobacteriaceae* (Gevers et al., 2014) increased (Figure 9B).

More taxa that produce the short chain fatty acid (SCFA) butyrate are increased in the HK-treated females than in the live-treated (Species known to be or likely to be capable of butyrate production are shown in orange; Figures 9B,C). Two of these species, *Clostridium symbiosum* and *Clostridium saccharobutylicum* increased in all 8 HK-treated animals with a large log_2_fc (Figure 9C). Also striking in the HK-treated females, is the increase in 7/8 animals in the actinobacterial taxa *Corynebacterium* and *Slackia heliotrinireducens* (Figure 9C). The species *Porphyromonas uenonis*, which can be pathogenic in humans (Finegold et al., 2004), decreased in HK-treated females. Family-level changes in HK-treated females include increases in the beneficial *Bifidobacteriaceae* family and the *Acholeplasmataceae* and *Thermoanaerobacteraceae* families and in the lower abundance *Acidaminococcaceae, Sporomusaceae, and Coriobacteriaceae* families. Decreasing in 6/8 HK-treated females is the *Sedimentibacter*.

The only taxon that differed in post-treatment relative abundance between HK- and live-treated females by whole-group analysis was *Capnocytophaga canis* (Figure 9D; Supplementary Table S21), which was due to a decrease in HK-treated females (Figure 9A).

### *L. reuteri* treatment correlates with specific differences in behavior, neurochemical expression, and bacterial taxa

We performed Spearman Rho correlations to examine significant associations across behavior, neurochemical expression, and *L. reuteri* abundance and fold change (Table 2). Our rationale for performing correlations with pre- to post-treatment fold change of *L. reuteri* as well as with post-treatment abundance was that each animal would be adjusted to its baseline level of *L. reuteri* and a large fold change in this species could modulate other phenotypes. In males, we found a negative correlation between CRF in the AMY and time in the OF center and a positive correlation between CRF in the AMY and post-treatment *L. reuteri* levels (Table 2). Also in males, there was a positive correlation between SA interaction and CRF in the NAcc. In females, we found a positive correlation between CRFR2 in the NAcc and V1aR in the PVN, whereas CRFR2 in the NAcc had a negative correlation with a pre- to post-treatment fold increase in *L. reuteri*. Somewhat surprisingly, anxiety-like behaviors in the SA test were negatively correlated with time in the closed arms of the EPM (Table 2).

**Table 2:** Correlations between brain neurochemical markers, behavioral phenotypes, and *L. reuteri* quantification.

Neurochemical marker expression or *L. reuteri* quantification	CRF, AMY	CRFR2, NAcc	OF center(s)	EPM open(s)	SA interaction	SA anxiety
**Female all**
V1aR, PVN		0.7				
OF center(s)						
EPM closed(s)				−1.0		−0.7
*L. reuteri* fold-change		−0.7				
**Male all**
CRF, AMY			−0.6			
CRF, NAcc					0.6	
*L. reuteri* post-treatment	0.7					

(*p* < 0.05 shown).Red color means a negative correlation. Green color means a positive correlation.

We also performed Spearman Rho correlations to identify interactions between other members of the stool microbiome and neurochemical markers and behaviors (Table 3). By the same rationale described above for *L. reuteri* itself, we correlated the neurochemical markers and behaviors with both the post-treatment abundance of the taxa and the log2fc of the taxa from pre-treatment to post-treatment. We used a method that takes into account the compositional nature of microbiome data to establish confidence intervals for correlations between the overall microbiome structure and the brain and behavior phenotypes and the *L. reuteri* qPCR results (Shi et al., 2016). In this method, if no correlation is found between a phenotype and the microbial community as a whole, the correlations with individual taxa are rejected. In the female 16S rRNA gene results, two of the most notable associations were positive correlations between the percent time spent in social interaction and increases in a *Ruminococcaceae* UCG-014 uncultured bacterium and in a *Ruminococcaceae* UCG-010 uncultured organism (Table 3). These taxa feature in additional results. This same *Ruminococcaceae* UCG-014 uncultured bacterium increased in HK-treated females (Figure 7), and decreased in live-treated males (Figure 7) and HK-treated males (Figure 6). A different unidentified *Ruminococcaceae* UCG-014 negatively correlated with time in the open arms of the EPM. In females, a different *Ruminococcaceae* UCG-010 metagenome species positively correlated with the OF center. A third *Ruminococcaceae* UCG-010 species positively correlated with post-treatment abundance of *L. reuteri* in females. In males, a fourth *Ruminococcaceae* UCG-010 uncultured bacterium negatively correlated with interaction in the SA test and positively correlated with time in the corner in the SA test.

**Table 3:** Microbial taxa detected by 16S rDNA V3-V4 analysis correlate with neurochemical marker expression in the brain and behavioral phenotypes.

16S V3-V4 most precise taxa defined	*L. reut* post qPCR	*L. reut* fc qPCR	CRF AMY	CRF NAcc	CRF PVN	V1aR PVN	CRFR2 NAcc	OF center(s)	EPM open(s)	EPM Closed(s)	SA inter-action	SA corner	SA anxiety
**Females**													
*L. reut* fc, qPCR							−0.7						
*Desulfovibrio* (log2fc)						0.6	0.6						
*Alphaproteobacteria* bacterium canine oral taxon 081 (log2fc)							0.7						
*Millionella massiliensis* (log2fc)							0.6						
*Allobaculum* gut metagenome (log2fc)							−0.7						
*Ruminococcaceae* UCG-010 metagenome (log2fc)								0.7					
*Treponema* 2 (post)	0.7												
*Ruminococcaceae* UCG-010 (post)	0.7												
*Ruminococcus* 1 uncultured bacterium (post)	0.7												
*Desulfovibrio* uncultured bacterium (post)	0.7												
*Gastranaerophilales* uncultured bacterium (log2fc)		−0.6											
*Rickettsiales* uncultured rumen bacterium (log2fc)		−0.6											
*Mollicutes* RF39 uncultured bacterium (post)			−0.7										
*Clostridiales* vadinBB60 group uncultured rumen bacterium (post and log2fc)			−0.7										
*Lachnospiraceae* (post)			0.7										
*Prevotellaceae* UCG-001 (post)				0.6									
*Christensenellaceae* uncultured (log2fc)				0.7									
*Bacteroidia* (post)					−0.7								
*Elusimicrobium* uncultured bacterium (post)									0.7	−0.7			
*Ruminococcaceae* UCG-014 unidentified (log2fc)									−0.6				
*Ruminococcaceae* UCG-014 uncultured bacterium (log2fc)											0.8		
*Ruminococcaceae* UCG-010 uncultured organism (log2fc)											0.8		
**Females family level**													
*Gastranaerophilales* uncultured bacterium (log2fc)		−0.7											
*Rickettsiales* uncultured (log2fc)		−0.7											
**Males**													
*L. reut* post, qPCR			0.7										
*Rikenellaceae* RC9 gut group, uncultured bacterium (post)	0.7												
*Lachnospiraceae* NK4A136 group (log2fc)		0.7											
*Lachnospiraceae* NK4A136 group (post)					−0.6				−0.6				
*Coprococcus* uncultured bacterium (log2fc)				0.6									
*Bacteroidales* p-251-o5 uncultured bacterium (post)					−0.7								
*Bacteroidales* p-251-o5 uncultured bacterium (log2fc)											0.6		
*Eubacterium ruminantium* group uncultured bacterium (post)					−0.6							0.7	
*Lachnospiraceae* NK4A136 group *Clostridiales* bacterium CIEAF 020 (log2fc)						0.7							
*Treponema* 2 (post)						−0.7				0.7			
*Treponema* 2 (log2fc)							−0.6						
*Clostridiales* vadinBB60 group (post)							−0.6				0.7		
*Clostridiales* vadinBB60 group uncultured rumen bacterium (log2fc)							−0.6						
*Clostridiales* vadinBB60 group uncultured rumen bacterium (post)												0.7	
*Desulfovibrio* (log2fc)							−0.7						
*Alistipes* uncultured bacterium (log2fc)									−0.7				
*Alistipes* uncultured bacterium (post)												0.7	
*Ruminiclostridium* 6 uncultured bacterium (post)									−0.6				
*Bacteroidia* (post)									−0.8	0.7			
*Eubacterium coprostanoligenes* group (log2fc)									−0.6	0.6			
*Eubacterium coprostanoligenes* group uncultured bacterium (log2fc)									0.8	−0.7			
*Allobaculum* gut metagenome species (post)									0.6	−0.7			
*Gastranaerophilales* uncultured rumen bacterium (post)										0.7			
*Oscillibacter* sp. 1–3 (log2fc)											0.7		
*Sphingobacteriaceae* (log2fc)											0.7		
*Ruminococcaceae* UCG-010 uncultured bacterium (post)											−0.8		
*Ruminococcaceae* UCG-010 uncultured bacterium (log2fc)												0.6	
*Bacteroidales* Rs E47 termite group uncultured bact. (post)													−0.8
**Males family level**													
*Rikenellaceae* (post)	0.6								−0.8	0.6			
*Helicobacteraceae* (post)									−0.7	0.8			
*Bacteroidia* (post)									−0.8	0.8			
*Spirochaetaceae* (post)									−0.8	0.8			
*Gastranaerophilales* uncultured rumen bacterium (post)										0.7			
*Erysipelotrichaceae* (post)									0.7	−0.7			
*Sphingobacteriaceae* (log2fc)											0.7		
*Bacteroidales* Rs E47 termite group (post)													−0.8

(*p* ≤ 0.01 shown).Red color means a negative correlation. Green color means a positive correlation.

Correlations between taxa and neurochemical markers in females include the *Desulfovibrio* genus, which positively correlated with both V1aR in the PVN and CRFR2 in the NAcc (Table 3). Also in females, a *Desulfovibrio* uncultured bacterium positively correlated with post-treatment *L. reuteri* level (Table 3). In contrast, the *Desulfovibrio* genus negatively correlated with CRFR2 in the NAcc in males and was increased in post-live-treated males (Figure 6). Also in females, two additional taxa positively correlated with CRFR2 in the NAcc: an *Alphaproteobacteria* bacterium canine oral taxon 081 species, and *Millionella massiliensis* (Table 3). This *Alphaproteobacteria* bacterium also increased in 6/8 HK-treated females (Figure 7). An *Allobaculum* gut metagenome species (*Erysipelotrichaceae* family) negatively correlated with CRFR2 in the NAcc in females. In males, this same species positively correlated with time in the open arms of the EPM and increased post-treatment in 5/8 HK-treated males (Figure 7). There are several associations in females between taxa and CRF levels. CRF in the NAcc in females positively correlated with log2fold-increase of a *Christensenellaceae* uncultured species and with post-treatment abundance of a *Prevotellaceae* UCG-001 species (Table 3). This *Christensenellaceae* uncultured species also decreased post-treatment in both live- and HK-treated females (Figure 6). CRF in the PVN was the only neurochemical marker that was higher in live-treated than in HK-treated females (Figure 3), and it had a negative correlation with post-treatment abundance of a *Bacteroidia* species (Table 3). This *Bacteroidia* species increased in 5/8 HK-treated females (Figure 7). In males, it positively correlated with time in the closed arms of the EPM (Table 3). Additional positive correlations in females were between *L. reuteri* post-treatment abundance and *Treponema* 2 and *Ruminococcus* 1 uncultured bacterium (Table 3). The same *Treponema* 2 negatively correlated with V1aR in the PVN and CRFR2 in the NAcc in males, and positively correlated with time in the EPM closed arms in males (Table 3). At the family level in females, pre- to post-treatment fold increase of *L. reuteri* negatively correlated with *Gastranaerophilales* uncultured bacterium and *Rickettsiales* uncultured rumen bacterium (Table 3).

There were a large number of correlations between taxa and neurochemical markers and/or behaviors in males, some of which were already mentioned above in conjunction with the correlations in females. Interestingly CRF in the AMY, the one neurochemical marker that was significantly higher in live-treated males, correlated with abundance of *L. reuteri* and no other taxa. Another correlation with *L. reuteri* in males was a positive correlation between post-treatment abundance and the *Rikenellaceae* RC9 gut group uncultured bacterium and also with the *Rikenellaceae* family. The *Rikenellaceae* RC9 gut group uncultured bacterium increased in 6/8 live-treated males (Figure 7). There was also a positive correlation between *L. reuteri* fold change and an increase in *Lachnospiraceae* NK4A136 group species in males (Table 3). The *Lachnospiraceae* NK4A136 group species also increased in 6/8 live-treated males (Figure 7), and its post-treatment abundance negatively correlated with CRF in the PVN and with time in the open arms of the EPM in males (Table 3). There are several other taxa that link a neurochemical marker with a behavior in males. A *Clostridiales* vadinBB60 group species negatively correlated with CRFR2 in the NAcc and positively correlated with SA interaction (Table 3). The log2fc of a different member of the *Clostridiales* vadinBB60 group (uncultured rumen bacterium) also negatively correlated with CRFR2 in the NAcc and its post-treatment abundance positively correlated with SA corner time (Table 3). Also in males, an *Eubacterium ruminantium* group uncultured bacterium negatively correlated with CRF in the PVN and its log2fc increase positively correlated with SA corner (Table 3). The post-treatment abundance of *Bacteroidales* p-251-o5 uncultured bacterium negatively correlated with CRF in the PVN and its log2fc positively correlated with SA interaction (Table 3). This family also increased in 5/8 live-treated males (Figure 7). An increase in *Alistipes* uncultured bacterium negatively correlated with time in the EPM open arms and its post-treatment abundance positively correlated with time in the SA corner (Table 3). Three taxa correlated with SA interaction in males, but with no neurochemical markers. The *Ruminococcaceae* UCG-010 uncultured bacterium mentioned above negatively correlated with SA interaction and its post-treatment increase positively with SA corner. An *Oscillibacter* sp. 1–3 species, a *Sphingobacteriaceae* species and the *Sphingobacteriaceae* family as a whole positively correlated with SA interaction in males (Table 3). A *Bacteroidales* Rs E47 termite group uncultured bacterium negatively correlated with anxiety in the SA test (Table 3).

In the WGS taxa correlations (females only), increases in two species, *Bifidobacterium* sp. AGR2158 and *Pontibacillus yanchengensis* had both a negative association with *L. reuteri* fold change and a positive association with V1aR in the PVN (Table 4). *Bifidobacterium* sp. AGR2158 increased in a majority of HK-treated females (Supplementary Table S20) and decreased in a majority of live-treated females (Supplementary Table S19). The *Bifidobacteriaceae* family positively associated with time in the OF center (Table 4), and it increased in 7/8 HK-treated females (Figure 9C). The strongest positive association between an increase in *L. reuteri* and a species was with *Streptococcus agalactiae* (Table 4). *S. galactiae* is unlikely to have been administered as a contaminant with *L. reuteri* since this species was detected in pre-treatment samples and was not detected in 2 separate aliquots of live *L. reuteri* for which the 16S rRNA gene V3-V4 amplicon sequences were determined (Supplementary Figure S1). At the family level, final abundance of *L. reuteri* positively correlated with *Helicobacteraceae, Sporolactobacillaceae*, and *Candidatus* Methanomethylophilaceae. Interestingly, an increase in *Helicobacteraceae* and the *Campylobacteriales* order overall positively associated with CRF in the NAcc (Table 4). There were no associations at the species level with CRF in the NAcc, but at the family level it also positively correlated with *Acholeplasmataceae* and *Aquificaceae* and negatively correlated with *Propionibacteriaceae* (Table 4). The *Acholeplasmataceae* increased in 7/8 of HK-treated females (Figure 9C). CRF in the PVN positively correlated with 5 *Prevotella* species and the *Prevotella* genus overall (Table 4). One of these species, *Prevotella* sp. S7-1-8, decreased in a majority of HK-treated females (Figure 9C).

**Table 4:** Microbial taxa detected by WGS analysis correlate with neurochemical marker expression in the brain and behavioral phenotypes.

WGS most precise taxa defined	*L. reut* post qPCR	*L. reut* fc qPCR	CRF AMY	CRF NAcc	CRF PVN	V1aR PVN	CRFR2 NAcc	OF center(s)	EPM open(s)	EPM closed(s)	SA inter-action	SA corner	SA anxiety
**Females**													
*Dyadobacter* sp. Leaf189 (log2fc)		−0.7											
*Bifidobacterium* sp. AGR2158 (log2fc)		−0.6				0.8							
*Pontibacillus yanchengensis* (log2fc)		−0.7				0.7							
*Cellulophaga* (log2fc)		−0.7											
*Dokdonia* sp. PRO95 (log2fc)		−0.7											
*Mesoaciditoga lauensis* (log2fc)		−0.6											
*Paenibacillus sp. JDR 2* (log2fc)		−0.7											
*Tepidanaerobacter acetatoxydans* (log2fc)		−0.7											
*Anaerosalibacter* sp. ND1 (*A. massiliensis*) (log2fc)		0.7											
*Ruminococcus* sp. JC304 (log2fc)		0.7											
bacterium LF-3 (log2fc)		0.6											
*Bergeyella zoohelcum* (log2fc)		0.7											
*Clostridiaceae bacterium* MS3 (log2fc)		0.7											
*Clostridium* sp. FS41 (log2fc)		0.7											
*Desulfatirhabdium butyrativorans* (log2fc)		0.7											
*Oscillospiraceae* (log2fc)		0.6											
*Paenibacillus* sp. VKM B 2647 (log2fc)		0.7											
*Streptococcus agalactiae* (log2fc)		0.8											
*Treponema phagedenis* (log2fc)		0.6											
*Prevotella* sp. MA2016 (post)					0.6								
*Bacteroides* sp. 3 1 40A (post)					0.6								
*Candidatus* Azobacteroides pseudotrichonymphae (post)					0.7								
*Clostridium homopropionicum* (post)					0.6								
*Clostridium sulfidigenes* (post)					0.7								
*Methanobrevibacter oralis* (post)					0.8								
*Porphyromonas bennonis* (post)					0.7								
*Prevotella* (post)					0.6								
*Prevotella maculosa* (post)					0.6								
*Prevotella* sp. KHD1 (post)					0.7								
*Prevotella* sp. MSX73 (post)					0.7								
*Prevotella* sp. S7-1-8 (post)					0.7								
*Shuttleworthia* sp. MSX8B (post)					0.7								
*Thioalkalivibrio* (post)					0.6								
*Clostridium acetobutylicum* (post)					−0.6								
*Desulfovibrionales* (post)					−0.6								
*Thermophagus xiamenensis* (post)					−0.8								
*Ulvibacter* sp. LPB0005 (post)					−0.7								
*Prevotella histicola* (log2fc)						−0.7							
*Clostridium* sp. LF2 (log2fc)						−0.7							
*Clostridium* sp. Marseille P2434 (log2fc)						−0.7							
*Solobacterium moorei* (log2fc)						−0.6							
*Bacteroidales bacterium* Barb6 (log2fc)						0.7							
*Bacillus* sp. X1 2014 (log2fc)						0.7							
*Bacteroidetes* bacterium oral taxon 272 (log2fc)						0.7							
*Clostridium saccharolyticum* (log2fc)						0.6							
*Bacillus methanolicus* (log2fc)						0.6							
*Clostridiales* bacterium SIT13 (log2fc)						0.7							
*Gemella* (log2fc)						0.6							
*Geobacter* (log2fc)						0.7							
*Heliobacterium modesticaldum* (log2fc)						0.7							
*Prevotella* sp. F0091 (log2fc)						0.7							
*Ruminococcus bicirculans* (log2fc)						0.8							
*Sharpea azabuensis* (log2fc)						0.6							
*Vibrio* (log2fc)						0.6							
**Females family level**													
*Candidatus* Methanomethylophilaceae (post)	0.7												
*Sporolactobacillaceae* (post)	0.8												
*Helicobacteraceae* (post)	0.7												
*Helicobacteraceae* (log2fc)				0.7									
*Campylobacteriales* (log2fc)				0.7									
*Acholeplasmataceae* (log2fc)				0.7									
*Aquificaceae* (log2fc)				0.7									
*Propionibacteriaceae* (log2fc)				−0.7									
*Halomonadaceae* (log2fc)							−0.7						
*Thermosediminibacteraceae* (log2fc)							−0.7						
FCB group (log2fc)							−0.8						
*Actinobacteria* (phylum) (log2fc)							0.6						
*Tepidanaerobacteraceae* (log2fc)								0.7					
*Bifidobacteriaceae* (log2fc)								0.7					
*Brachyspiraceae* (log2fc)								0.7					
*Chitinispirillaceae* (log2fc)								0.7					
*Endozoicomonadaceae* (log2fc)								0.7					
*Micromonosporaceae* (log2fc)								0.7					
*Paludibacteraceae* (post)									−0.6				
*Thermaceae* (post)									−0.7				
*Pirellulaceae* (post)									−0.6				
*Leuconostocaceae* (post)									−0.6				−0.7
*Sulfurovaceae* (post)													−0.7
*Xenococcaceae* (post)													−0.7
*Rhodobacteraceae* (post)													0.7

(*p* ≤ 0.01 shown).Red color means a negative correlation. Green color means a positive correlation.

The strongest positive correlation for CRF in the PVN was with post-treatment abundance of *Methanobrevibacter oralis* and the strongest negative correlation was with *Thermophagus xiamenensis* (Table 4). The strongest positive association between V1aR in the PVN and a taxon other than *Bifidobacterium* sp. AGR2158 was *Ruminococcus bicirculans* (Table 4). There were no associations at the species level with CRFR2 in the NAcc, but at higher taxonomic levels there was a positive correlation with the *Actinobacteria* and there were negative correlations with the FCB group and the *Halomonadaceae* and *Thermosediminibacteraceae* families (Table 4). The correlations between taxa in the WGS and behavior are not straightforward. Strikingly, at the WGS species level, there were no associations between taxa and any behaviors (Table 4), because social interaction was not found to have an association with the microbiome as a whole. However, 4 families had associations with SA anxiety. The *Rhodobacteriaceae* were positively correlated with SA anxiety (Table 4). The *Leuconostocaceae, Sulfurovaceae*, and the *Xenococcaceae* were negatively correlated with SA anxiety. Counterintuitively, the *Leuconostocaceae* were also negatively correlated with the open arms of the EPM, but this is consistent with the negative correlation between SA anxiety and time in the EPM closed arms (Table 2). Additional families negatively correlated with time in the EPM open arms are *Paludibacteraceae, Thermaceae*, and *Pirellulaceae*. In addition to the *Bifidobacteriaceae*, the families *Tepidanaerobacteraceae*, *Brachyspiraceae, Chitinispirillaceae, Endozoicomonadaceae*, and *Micromonosporaceae* were positively correlated with time in the OF center (Table 4).

## Discussion

Probiotic intake has been shown to alter behaviors *via* the gut-brain axis both in humans and in traditional rodent models (Messaoudi et al., 2011; Ait-Belgnaoui et al., 2014; Liu et al., 2015; Bermúdez-Humarán et al., 2019). In the present study, our data indicate that intake of *L. reuteri* altered affiliative behaviors in a sex-specific manner in socially monogamous prairie voles, supporting the notion that *L. reuteri* affects social behavior (Buffington et al., 2016; Mackos et al., 2016; Sgritta et al., 2019; Sherwin et al., 2019). In addition, our data show effects of live *L. reuteri* intake on neurochemical and neurochemical receptor expression, microbiome composition, and significant correlations between these alterations in the gut, neurochemical expression and behavior. Taken together, these data further illustrate the role of the gut-brain axis in the regulation of social behaviors (Arentsen et al., 2015; Sylvia and Demas, 2018; Sgritta et al., 2019; Sherwin et al., 2019; Donovan et al., 2020b).

Our behavioral data are interesting in several aspects. First, although data from traditional rodent models have shown a prosocial effect of live *L. reuteri* (Buffington et al., 2016; Sgritta et al., 2019), our data indicate that intake of live *L. reuteri* resulted in significantly less social interaction in female prairie voles compared to HK-treated ones. Unlike traditional lab rodent species, the prairie vole is a socially monogamous species that naturally displays high levels of social affiliation toward conspecifics (Beery et al., 2018; Lee et al., 2019). Therefore, species differences may lead to differential responses to *L. reuteri* treatment. In addition, differences in experimental protocols, such as supplementation (the present study) vs. restoration (Buffington et al., 2016) of *L. reuteri*, may also result in differential impacts on social behavior. The sex-specific effect of significantly less social affiliation in live-*L. reuteri-*treated relative to HK-treated female, but not male voles, along with the sex-specific effects seen in neurochemical marker expression and microbiome composition (see below) is intriguing, as sex-specific neurochemical regulation of social behaviors have been reported in prairie voles (DeVries et al., 1995; Cho et al., 1999) as well as in rats and mice (Dumais et al., 2016; Kopec et al., 2018; Rigney et al., 2020). Although *L. reuteri* treatment has also been shown to alter stress-related behaviors in other species (Cryan and O'Mahony, 2011; Marin et al., 2017), we found no effects of *L. reuteri* on anxiety-like behavior in the present study. These data further indicate that *L. reuteri* can affect behaviors in a species- and behavior-specific manner. Further, our data from all behavioral assays consistently indicated lower levels of anxiety-like behaviors in females compared to males. These data are in agreement with that from previous studies demonstrating sex differences in anxiety-like behaviors across a variety of species, including humans (Donner and Lowry, 2013; Domonkos et al., 2017; Scholl et al., 2019).

Our data illustrate that live *L. reuteri* administration was also associated with altered neurochemical and receptor expression in a sex-, brain region- and neurochemical-specific manner. Fascinatingly, we found that females treated with live *L. reuteri* had higher expression of CRF in the PVN and lower CRF and CRFR2 in the NAcc compared to those treated with HK-control *L. reuteri*. Increased CRF in the PVN can lead to increased activity of the hypothalamic–pituitary–adrenal (HPA) axis, and this increased HPA activity has been previously shown to impair social contact/affiliation in female prairie voles (DeVries et al., 1995). Although not tested in females, CRF administration directly into the NAcc of male prairie voles facilitates social bond formation by acting on both CRFR1 and CRFR2 in the NAcc (Lim et al., 2007). Therefore, higher CRF expression in the PVN and lower CRF and CRFR2 expression in the NAcc may each or both be contributing to the lower social affiliation seen in live-*L. reuteri* treated female prairie voles. Our data also showed lower V1aR expression in the PVN in females treated with live *L. reuteri* compared to HK-treated females. These data are intriguing, as the brain AVP system serves as an important modulator for a variety of social behaviors (Murakami et al., 2011). In particular, PVN V1aR is highly correlated with social interaction (Murakami et al., 2011) and is directly involved in regulating mother-pup interactions (Bayerl et al., 2016). It is plausible that lower V1aR in the PVN plays a synergistic role with the CRF system in the lower level of social affiliation induced by live *L. reuteri* – a speculation that needs to be tested in subsequent studies. These effects of live *L reuteri* intake on the CRF and AVP systems in the female vole brain may indicate the potential underlying neurochemical basis by which *L reuteri* affects social affiliation in a sex-specific manner. In addition, our data support the notion that probiotic intake can affect multiple neurochemical systems in a brain circuitry underlying motivated behaviors in voles (Young et al., 2011b; Neumann and Landgraf, 2012; Hostetler and Ryabinin, 2013; Beurel and Nemeroff, 2014; Dumais and Veenema, 2016; Johnson and Young, 2017; Winter and Jurek, 2019; Piccin and Contarino, 2020).

To fully explore the role of specific members of the microbiome in the different treatment groups, we analyzed the representation of specific taxa by multiple methods. In the VBV WGS method, the increase in *Lactobacillus* detected in live-treated females was consistent with our qPCR results, providing support to the validity of this approach. Live *L. reuteri* treatment also induced changes (pre- to post-changes) in several taxa of gut microbiota. The most prominent increasing taxon in this group is *Clostridiales* bacterium VE202-01. This strain is part of a consortium of *Clostridia* being developed as a therapeutic for inflammatory bowel disease (Atarashi et al., 2013), and it has also been associated with diarrhea in immunocompromised mice (Misic et al., 2018). The proteobacterial family *Enterobacteriaceae,* also increased in a majority of live-treated females. *Enterobacteriaceae* are associated with new-onset Crohn’s disease (Gevers et al., 2014), and many species in this family are known for production of lipopolysaccharide (LPS) forms that are strong agonists for the TLR4 receptor that mediates LPS-induced inflammation (Steimle et al., 2016). Interestingly, when looking at the representation of *L. reuteri* and *Lactobacillus*, baseline differences were also found between male and female prairie voles. Prior to *L. reuteri* treatment, males had more *Lactobacillus* compared to females. The increase in *L. reuteri* abundance after treatment with live *L. reuteri* was much greater for males than for females. This was surprising, since the differences in social interaction and most of the differences in neurochemical markers in live-treated animals were observed in females. On the other hand, the overall structure of the microbiome was quite different between females and males, with greater bacterial taxa richness in females both at baseline and after treatment with live *L. reuteri*. It is not yet clear what impacts the higher baseline level of *Lactobacillus* in males or the greater taxa richness in females might have on behavior or neurochemical markers. Sex differences in microbiome composition and species richness have been previously found in human and other rodent models (Jašarević et al., 2016; Kim et al., 2020) – these results further suggest that *L. reuteri* may play differential roles in males and females.

Our correlation analyses indicate some interesting associations between gut microbiota alterations and behavior. For example, we found that an increase in *Ruminococcaceae* UCG-014 uncultured bacterium and in a *Ruminococcaceae* UCG-010 uncultured organism positively correlated with social affiliation in female prairie voles. In our previous study, an increase in a different *Ruminococcaceae* UCG-010 species (UCG-010 uncultured bacterium) was also strongly correlated with social affiliation in cohoused prairie voles (Donovan et al., 2020b). The literature on the effects of *Ruminococcaceae* UCG-010 on social behavior are equivocal. One study found that *Ruminococcaceae* UCG-010, unlike most other *Ruminococcaceae*, was less abundant in an autism rat model than in controls (Kong et al., 2021). In contrast, another study showed that *Ruminococcaceae* UCG-010 was more abundant in children with autism spectrum disorder (ASD; Ma et al., 2019). There is also ambiguity in the effects of *Ruminococcaceae* UCG-014 on social behavior. Although we found that an increase in the *Ruminococcaceae* UCG-014 uncultured bacterium was negatively correlated with social interaction in cohoused prairie voles (Donovan et al., 2020b), *Ruminococcaceae* UCG-014 is more abundant in rats with hydrocortisone-induced depression than in control ones (Cheng et al., 2018) and is increased in high-anxiety relative to low-anxiety mice (Jin et al., 2021). Future studies should assess causal relationships between *Ruminococcaceae* UCG-014 and UCG-010 with social behavior.

We also found significant correlations between microbial taxa and neurochemical marker expression. Taxa that correlated with neurochemical markers in females included *Methanobrevibacter oralis*, which had the strongest positive correlation with CRF in the PVN. The methanogenic archaea of the genus *Methanobrevibacter* are associated with both anorexia nervosa and multiple sclerosis (Jangi et al., 2016). There were also positive correlations between CRF in the PVN and multiple species of *Prevotella* in female voles, and an increase in the *Desulfovibrio* genus was positively correlated with V1aR in the PVN and CRFR2 in the NAcc. In prairie voles, *Desulfovibrio* increased after social isolation (Donovan et al., 2020b), and some links between *Desulfovibrio* and autism have also been suggested in humans (Finegold, 2011). However, in a different study, reduced symptoms in children with ASD were associated with persistent elevation of *Desulfovibrio* (Kang et al., 2017), suggesting a positive effect of *Desulfovibrio* on ASD. Taken together, our results indicate that a variety of microbial taxa are associated with neurochemical expression, thus emphasizing that there may be multiple taxa involved in shaping changes in neurochemical expression after probiotic intake.

In the present study, we chose to use HK *L. reuteri* as our control, as previous studies have found no effects of HK *L. reuteri* on social behavior (Buffington et al., 2016), and usage of HK-administration can control for effects seen from simply ingesting the bacterial biomass itself. Interestingly, some striking changes in pre- to post-treatment microbiota in HK-treated females in the *Bifidobacteriaceae* were detected only by WGS sequence analysis. The detection of an increase in *Bifidobacteria* by WGS, but not by 16S V3-V4 amplicon sequencing could be due to an underestimation of *Bifidobacteria* by the V3-V4 primer set (Kameoka et al., 2021). By the whole-group method, the α-amylase-rich species, *Bifidobacterium boum* (Milani et al., 2016) increased after treatment with HK *L. reuteri*. By the VBV method, the *Bifidobactereaceae* family increased in HK-treated females, and the species *Bifidobacterium* sp. AGR2158 increased in HK-treated females, but decreased in live-treated females. *Bifidobacterium* sp. AGR2158 increase also positively correlated with V1aR in the PVN and negatively correlated with an increase in *L. reuteri*. Although there is no direct evidence of *Bifidobacteriaceae* regulation of social behaviors, a recent meta-analysis found that *Bifidobacteria* are reduced and *Lactobacilli* are elevated in ASD patients (Liu et al., 2019). A clinical-trial study reported that microbial transfer of standard human gut microbiota (SHGM) to ASD children improved ASD symptoms and resulted in persistent elevation of *Bifidobacterium* (Kang et al., 2017). *Bifidobacteria* are also at lower abundance in multiple gastrointestinal diseases (O'Callaghan and van Sinderen, 2016), and some species are able to block the inflammatory effects of proteobacterial lipopolysaccharide (Riedel et al., 2006). *Bifidobacteria* have also been shown to engage in a syntrophic interaction with butyrate producers by providing them with the substrates acetate and lactate (Alessandri et al., 2019). The other species that increased after after treatment with HK *L. reuteri*, *Blautia* sp. KLE 1732, has also been shown to be beneficial. It is among those associated with successful engraftment in *Clostridium difficile* patients after fecal microbiota transplantation from healthy donors (Verma et al., 2021). The same meta-analysis that found lower *Bifidobacteria* levels in ASD patients also found lower *Blautia* levels in that group (Liu et al., 2019).

A large number of butyrate-producing taxa (Kanauchi, 2006; Kelly et al., 2010; Scott et al., 2011; Van den Abbeele et al., 2013; Miguel et al., 2019; Palevich et al., 2020) also increased in abundance in females treated with HK *L. reuteri*. Butyrate produced in the gut is consumed as an energy source by intestinal epithelial cells and has additional beneficial effects that include induction of T regulatory cells in the intestinal mucosa and stimulation of serotonin secretion (Stilling et al., 2016). The butyrate producer, *Roseburia inulinivorans* is especially notable as the *Roseburia* have been shown to be lower abundance in people with inflammatory bowel disease (Zhu et al., 2018). Treatment with a *Roseburia* species has also been shown to reduce the effects of induced stress in rats (Zhang et al., 2019). Interestingly, butyrate has also been shown to facilitate social bonding in prairie voles (Wang et al., 2013) and attenuates social deficits in ASD mice (Kratsman et al., 2016). The increase in representation of butyrate-producing taxa indicate the possibility that butyrate may be involved with changes seen in the HK-treated females.

It should be noted that there is accumulating evidence showing that treatment with heat-killed *Lactobacillus* species may actually have beneficial effects. For example, in male mice that had experienced social defeat, HK-*L. helveticus* improved social interaction (Maehata et al., 2019). A mixture of HK *L. fermentum* and *L. delbrueckii* and their culture products also improved social interaction in male mice (Warda et al., 2019). Beneficial effects of HK preparations of other *Lactobacillus* species were found on the immune system and include enhancement of intestinal mucosa IgA antibody production (Arai et al., 2018; Pique et al., 2019). HK *Lactobacillus* can also have effects on the colonization properties of other members of the microbiome (Aiba et al., 2017; Pique et al., 2019). Inoculation with heat-killed material from some species of *Lactobacillus* increased the population of bifidobacterial species in both a human fecal fermentation model and in pure culture, and most of the bifidogenic material was associated with the cellular fraction (Warda et al., 2021). Our HK *L. reuteri* were prepared from frozen aliquots of cells that had been washed free of culture supernatant prior to freezing. Therefore, the factor(s) responsible for the effects we observed must also be *L. reuteri* cell-associated. Cell components responsible for these effects could be polysaccharide, protein, lipid or heat-stable metabolites made more accessible to other members of the gut community by prior heat killing. Material derived from the HK cells could also interact directly with the host gut epithelium as an innate immune or metabolic signal (Swanson et al., 2020).

Intriguingly, if we compare the behavioral data from the present study to those from a previous study in which social affiliation was assessed in cohoused prairie voles without *L*. *reuteri* treatment (Donovan et al., 2020b), the live-treated female affiliation time is comparable to the cohoused controls, whereas the HK-treated females show higher levels of affiliation. Thus, there is an intriguing possibility that the HK treatment may actually be prosocial. Although these comparisons must be taken with caution given differences in study design, this increase in sociality in HK-treated females could be related to availability of *L. reuteri* effectors, intracellular metabolites, or macromolecules, rather than effects that could only be provided by colonization with the live bacteria. Nevertheless, the potential beneficial effects of HK bacteria must be further explored in additional studies.

## Conclusion

Using the unique, socially monogamous prairie vole model, we found that intake of *L. reuteri* altered social behaviors and brain neurochemical marker expression in a behavior-, brain region-, and sex-specific manner. Inoculation with live *L. reuteri* also resulted in specific alterations to gut microbiota that differed across males and females, which were also associated with changes in the neurochemical systems and relevant behaviors. Further, our data also indicated the potential prosocial effects of HK *L. reuteri*. Together, these data have provided solid evidence to support the notion that multiple levels of sexual dimorphisms exist, from gut microbial alterations, to neurochemical circuits, to behaviors (Dumais and Veenema, 2016; Grabowska, 2016; Jašarević et al., 2016). Our data also highlight a discrepancy between 16S and WGS results, thus emphasizing the importance of including multiple types of microbial analyses in future studies. Taken together, our study further demonstrates the utility of the prairie vole model for studying the gut-brain axis and its regulation of social behaviors.

## Data availability statement

The datasets presented in this study can be found in online repositories. The names of the repository/repositories and accession number(s) can be found in the article/Supplementary material.

## Ethics statement

All procedures were approved by the Institutional Animal Care and Use Committee at Florida State University and were in accordance with the guidelines set forth by the National Institutes of Health.

## Author contributions

MD conceived the study. MD, CM, GP, JC, KJ, and ZW designed the study. MD, CM, GP, AB, BW, DT, YL, and KJ performed the experiments. MD, CM, ML, GP, AB, BW, TC, KJ, and ZW analyzed the data. MD, CM, KJ, and ZW wrote the manuscript. All authors contributed to the article and approved the submitted version.

## Funding

This work was supported by the National Institutes of Health (NIMH R01-108527, NIMH R01-109450, and NIMH R21-111998) to ZW; partially supported by the National Institute of Food and Agriculture, U.S. Department of Agriculture, award number 2014-67013-21579 to KJ; and MD was supported by the NIH program training grant (T32 MH093311, P. K. Keel and L. A. Eckel). For MD writing of manuscript was supported by the VA Office of Academic Affiliations, Advanced Fellowship Program in Mental Illness Research and Treatment, Department of Veterans Affairs.

## Conflict of interest

ML and TC were employed by Metagenom Bio Life Science Inc.

The remaining authors declare that the research was conducted in the absence of any commercial or financial relationships that could be construed as a potential conflict of interest.

## Publisher’s note

All claims expressed in this article are solely those of the authors and do not necessarily represent those of their affiliated organizations, or those of the publisher, the editors and the reviewers. Any product that may be evaluated in this article, or claim that may be made by its manufacturer, is not guaranteed or endorsed by the publisher.

## Footnotes

^1^ http://www.jwatcher.ucla.edu/

^2^ https://support.illumina.com/downloads/16s_metagenomic_sequencing_library_preparation.html

## Abbreviations

AMY: Amygdala
ASD: Autism spectrum disorder
AVP: Arginine vasopressin
CRF: Corticotrophin releasing factor
CRFR1: Corticotrophin releasing factor type-1 receptor
CRFR2: Corticotrophin releasing factor type-2 receptor
EPM: Elevated plus maze
GAPDH: Glyceraldehyde 3-phosphate dehydrogenase
HK: Heat-killed
HPA: hypothalamic pituitary–adrenal
LPS: Lipopolysaccharide
NAcc: nucleus accumbens
OT: Oxytocin
OTR: Oxytocin receptor
PERMANOVA: Permutational multivariate analysis of variance
PFC: Prefrontal cortex
PVN: Paraventricular nucleus of the hypothalamus
SHGM: Standard human gut microbiota
SDS: Sodium dodecyl sulfur
TBS: Tris buffered saline
VBV: Vole-by-vole
VTA: Ventral tegmental area
V1aR: Vasopressin 1a-receptor
