# Supplementary material for: Limosilactobacillus reuteri administration alters the gut-brain-behavior axis in a sex-dependent manner in socially monogamous prairie voles
Source: Front Microbiol. 2023 Feb 8;14:1015666. doi: 10.3389/fmicb.2023.1015666 (PMC9945313; doi:10.3389/fmicb.2023.1015666)
Supplement: Supplementary file 1 [file Data_Sheet_1.zip › Figures S1 - S6.PDF]

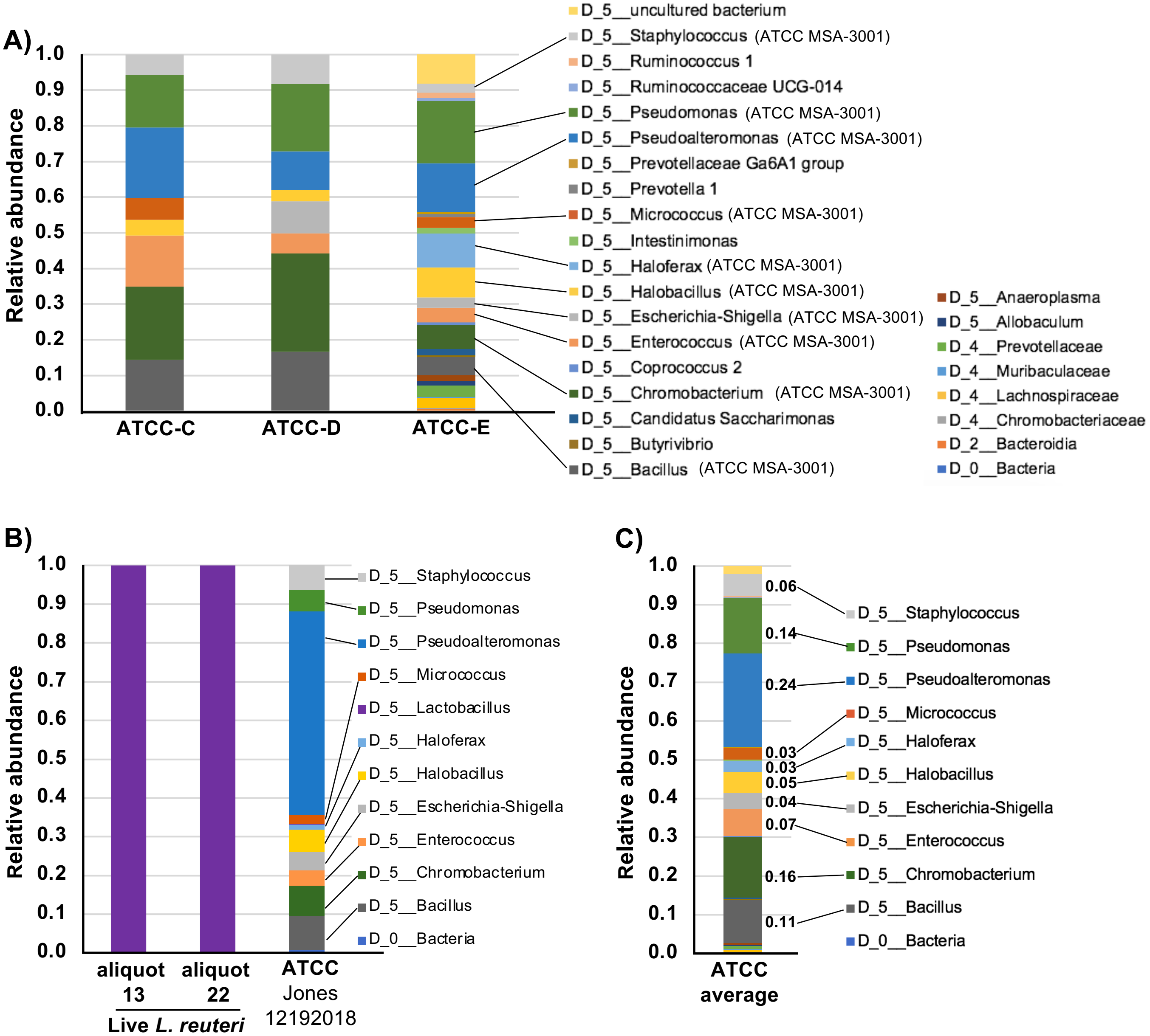

Figure S1. Relative abundance of bacterial taxa in the ATCC MSA-3001, ABRF-MGRG 10-Strain Even Mix Genomic Material and in aliquots of *L. reuteri* inoculum. A) ATCC-C was run on 16S rRNA gene V34 amplicon library 96-well plate C, which included the male vole samples shown in Table SX. ATCC-D was run on library plate D that included the male vole samples shown in Table SX. (The pre- and post-treatment samples for each animal were run on the same plate and the same number of live-treated and HK-treated samples were run on each plate.) ATCC-E was run on library plate E that included all female samples. Taxa not included in the ATCC MSA-3001 control were detected in ATCC-E. However, the no-template control for all library plates, including plate E, produced too little amplicon DNA to be detected by Qubit fluorimetry. Taxa that are included in ATCC MSA-3001 Strain Even Mix are noted in the legend. **B)** Aliquots 13 and 22 of *L. reuteri* administered to animals were sequenced and were determined to contain only *L. reuteri*. ATCC-Jones12192018 was run on the same library plate as the *L. reuteri* aliquots. **C)** Average of the 4 replicate V34 sequences of the ATCC control.

A) All, Sex difference

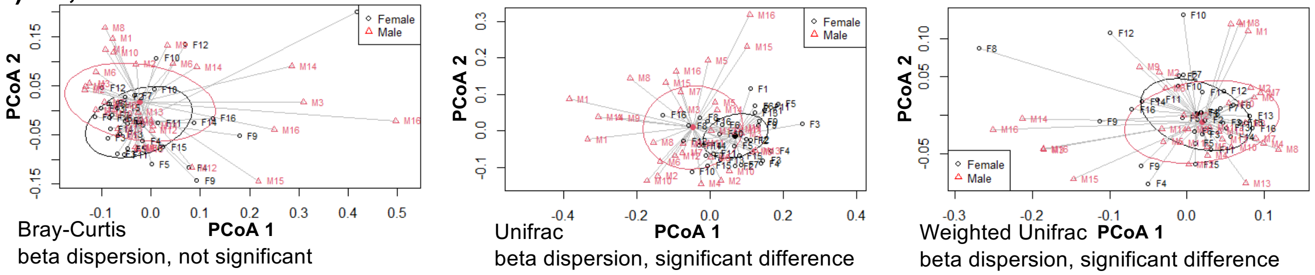

B) All, Probiotic treatment difference (HK-treated vs. Live-treated)

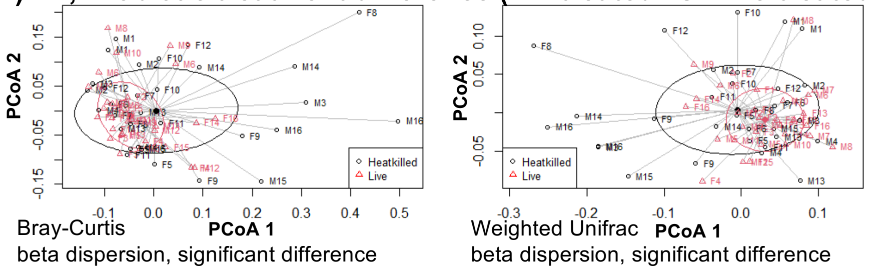

C) Female, Probiotic treatment difference (HK-treated vs. Live-treated)

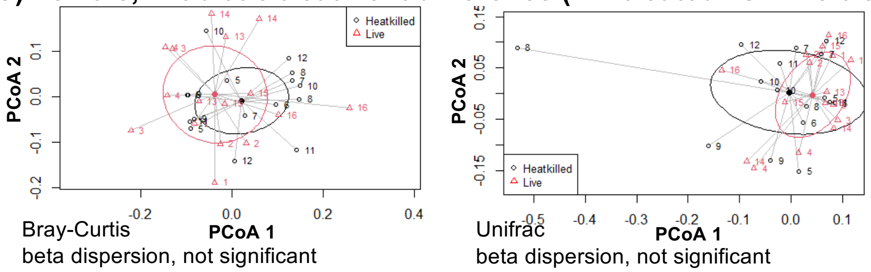

D) Pre-treatment, Sex difference

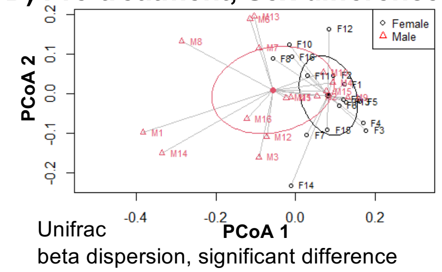

E) Post-treatment, Sex difference

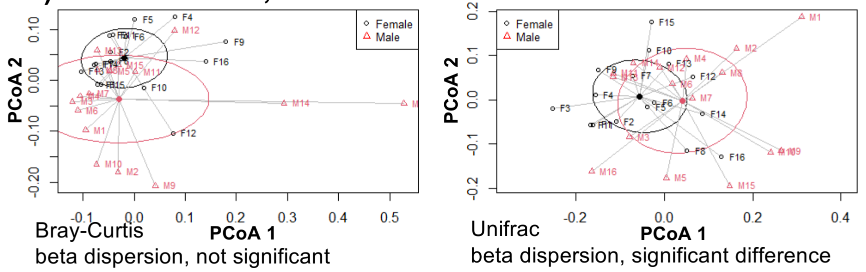

F) Heat-killed treated, Sex difference

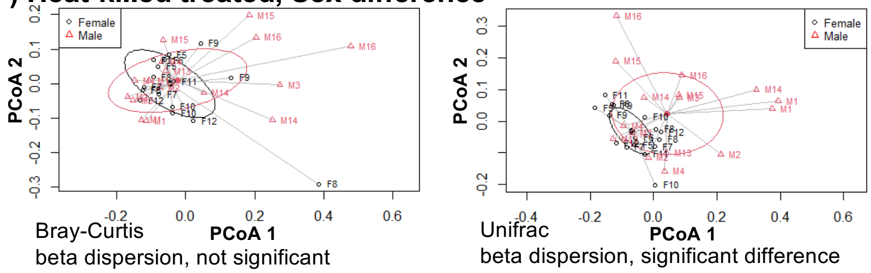

G) Live-treated, Sex difference

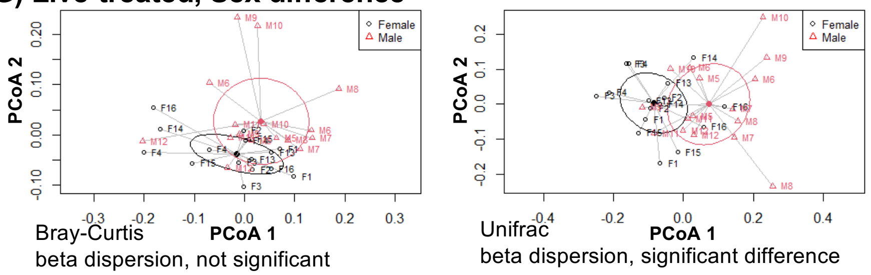

Figure S2. Differences in beta-dispersion in metrics with significant beta-diversity differences at the species level. The centroid of each group is show along with the distance between the centroid and each sample. The dispersion is shown by the circle. All letters correspond to a line in Table 1: A) Line1; B) Line 2; C) Line 4; D) Line 8; E) Line 10; F) Line 12; G) Line 14

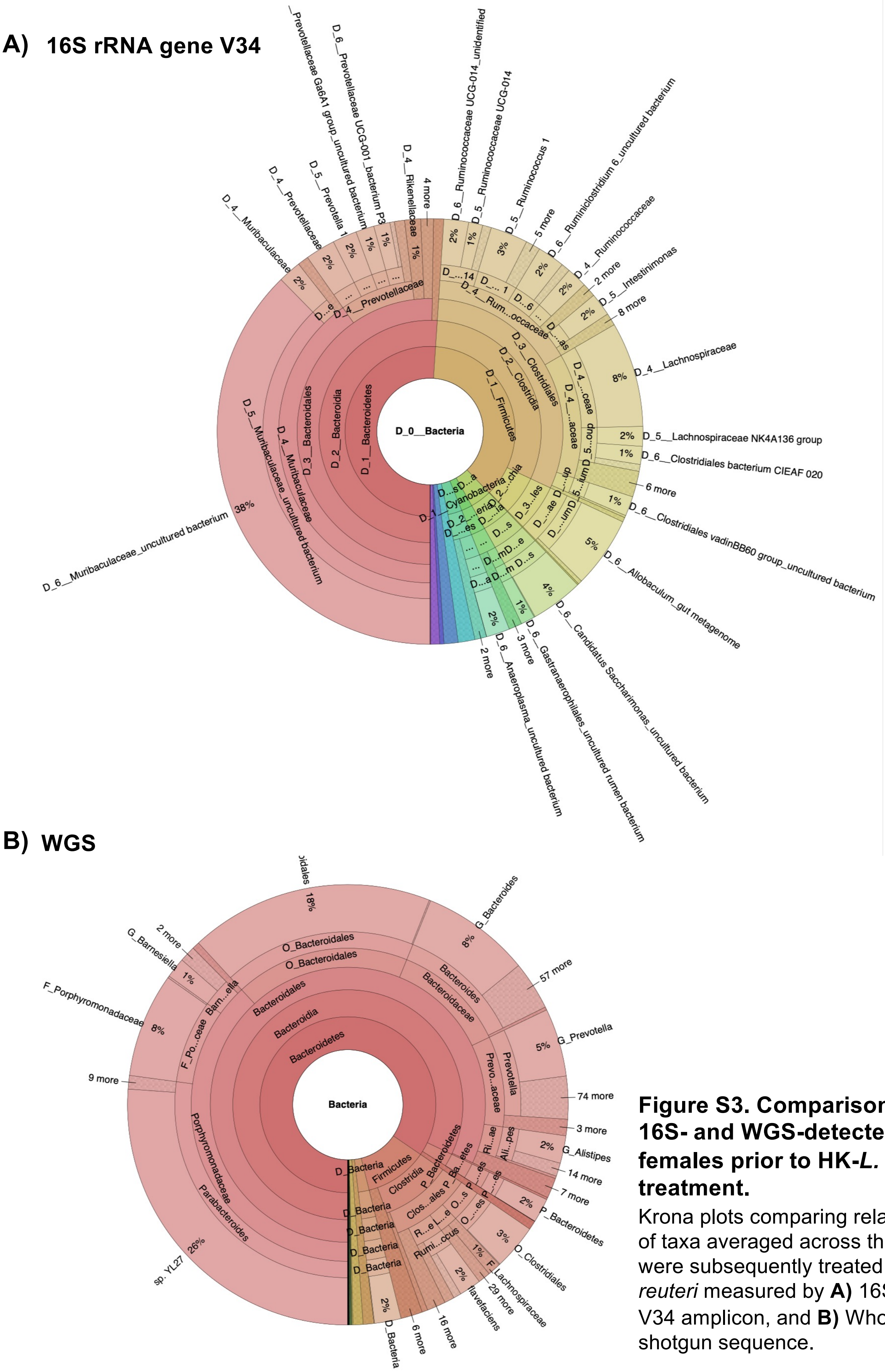

Figure S3. Comparison Krona plots of 16S- and WGS-detected taxa in females prior to HK-*L. reuteri* treatment.

Krona plots comparing relative abundance of taxa averaged across the 8 females that were subsequently treated with HK *L. reuteri* measured by **A)** 16S rRNA gene V34 amplicon, and **B)** Whole-genome shotgun sequence.

A) **16S rRNA gene V34**

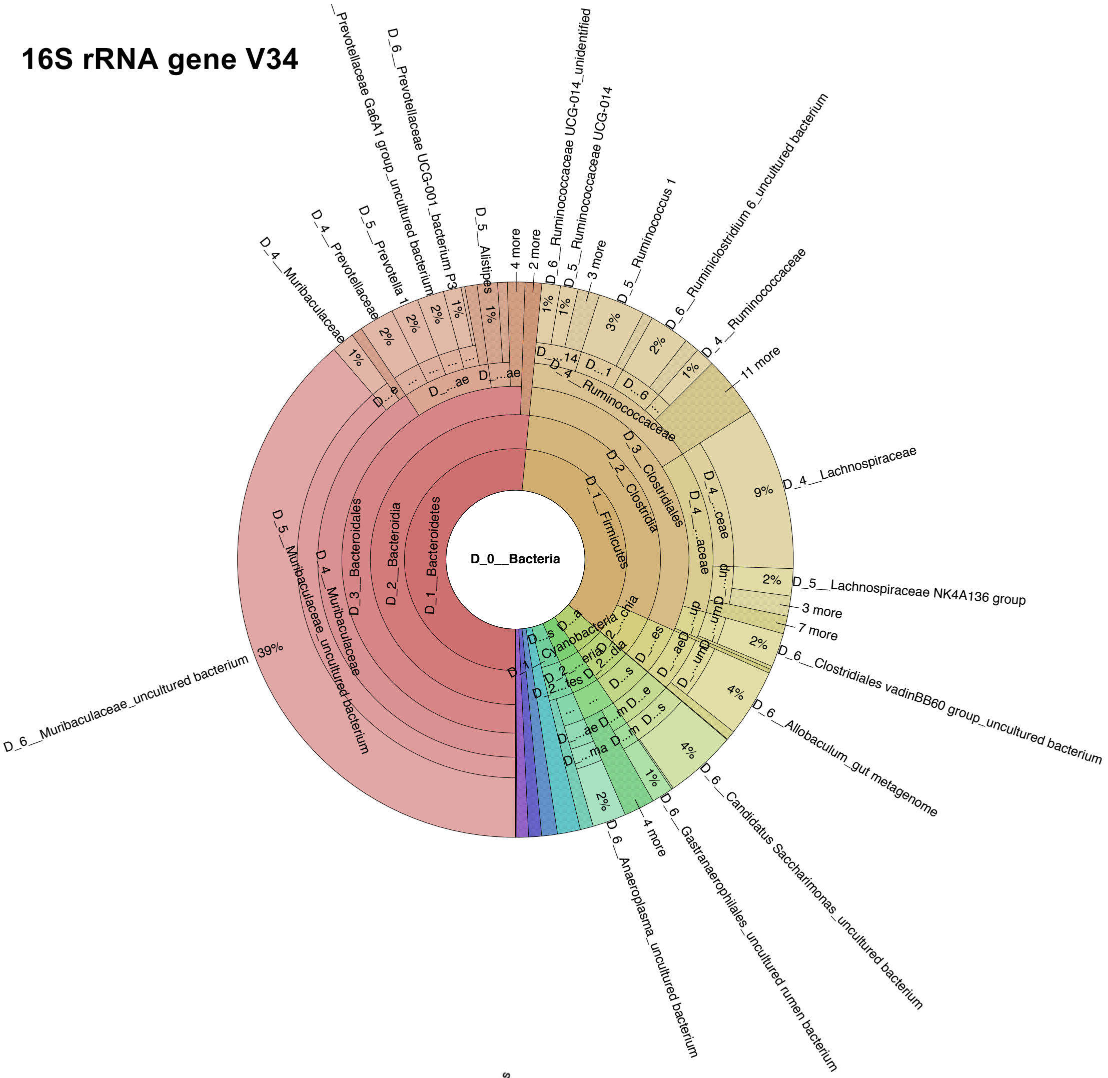

B) **WGS**

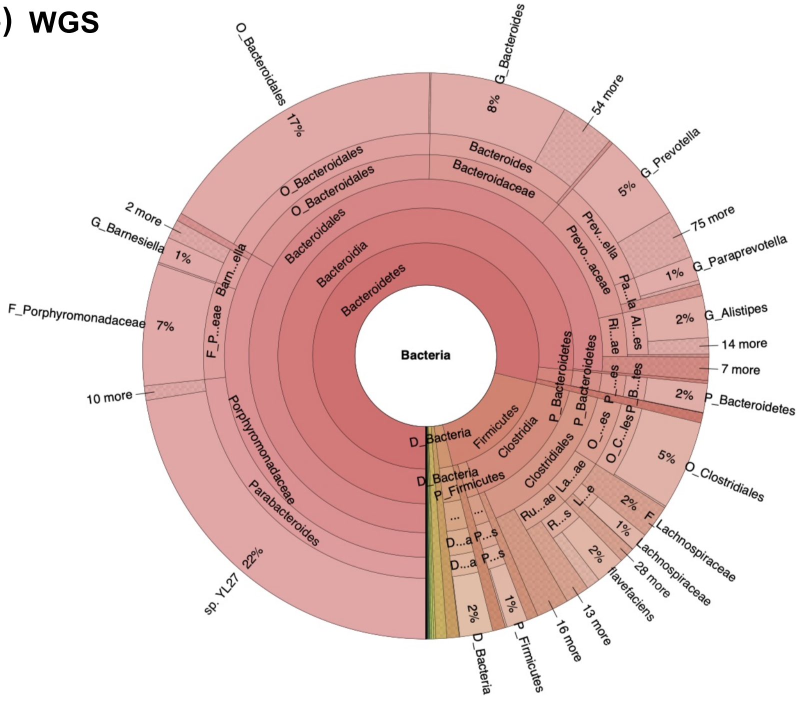

Figure S4. Comparison Krona plots of 16S- and WGS-detected taxa in females post-HK-*L. reuteri* treatment.
Krona plots comparing relative abundance of taxa averaged across the 8 HK-*L. reuteri*-treated females measured by **A)** 16S rRNA gene V34 amplicon, and **B)** Whole-genome shotgun sequence.

A) 16S rRNA gene V34

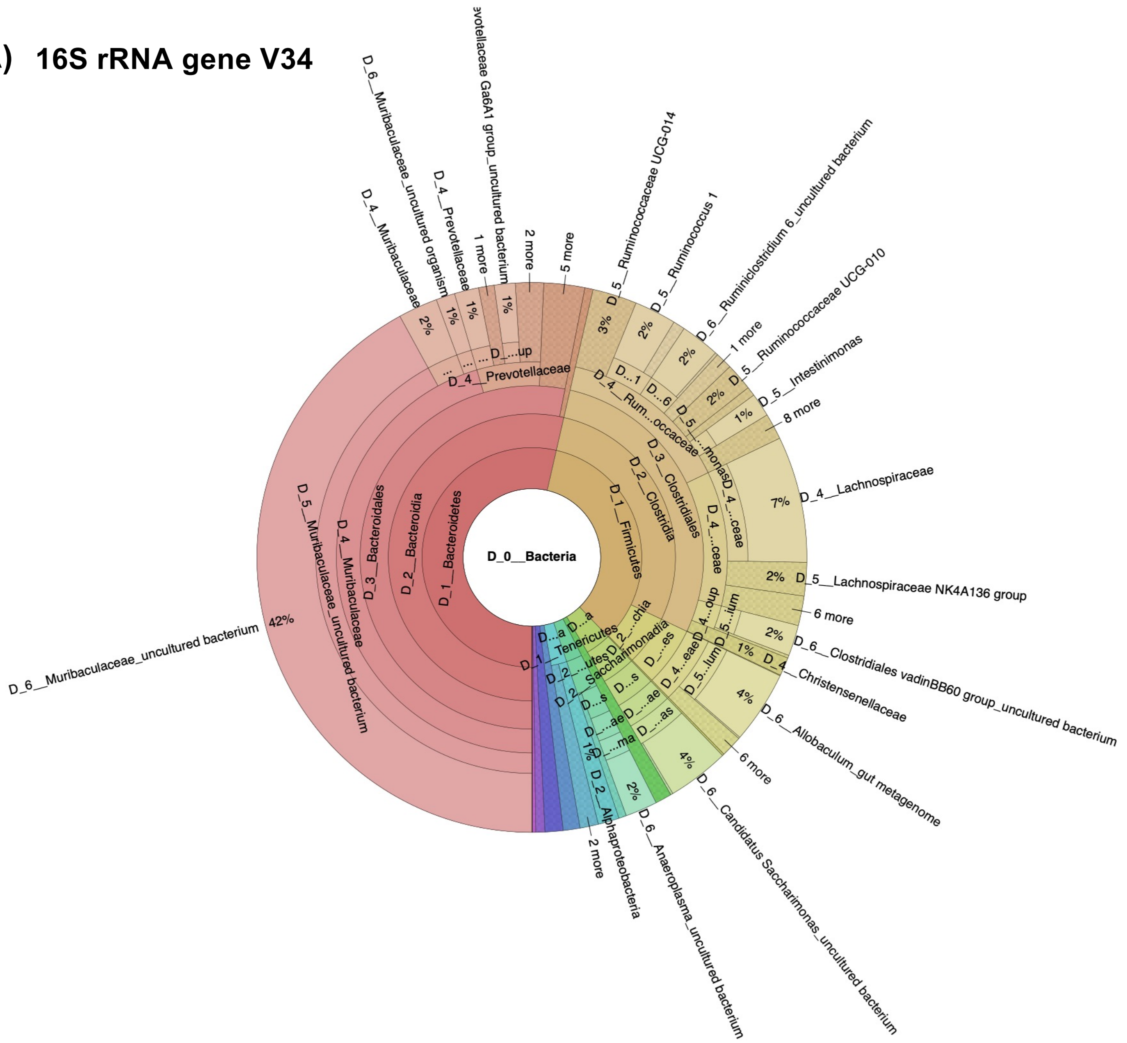

B) WGS

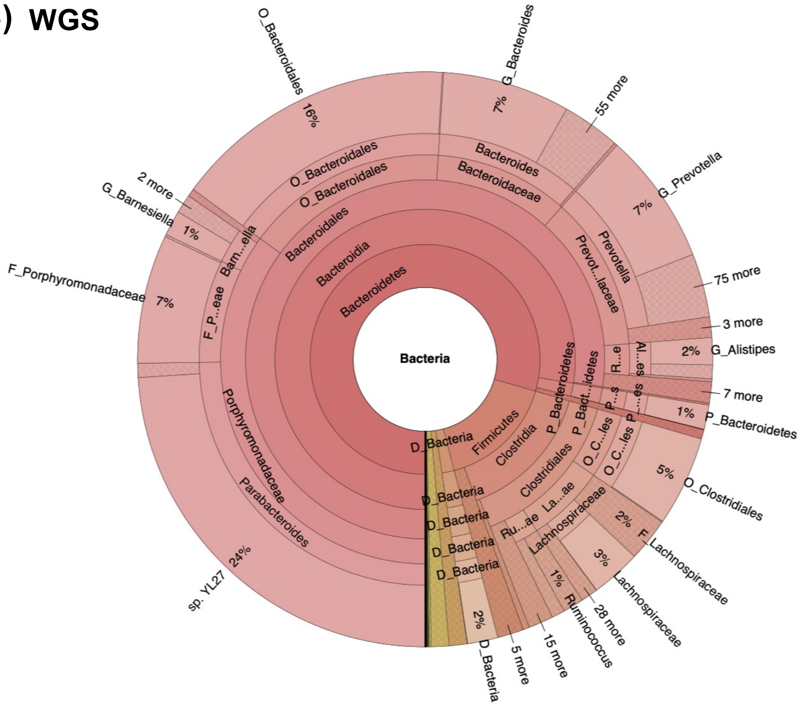

Figure S5. Comparison Krona plots of 16S- and WGS-detected taxa in females prior to live-*L. reuteri* treatment.
Krona plots comparing relative abundance of taxa averaged across the 8 females that were subsequently live-treated measured by **A)** 16S rRNA gene V34 amplicon, and **B)** Whole-genome shotgun sequence.

A) 16S rRNA gene V34

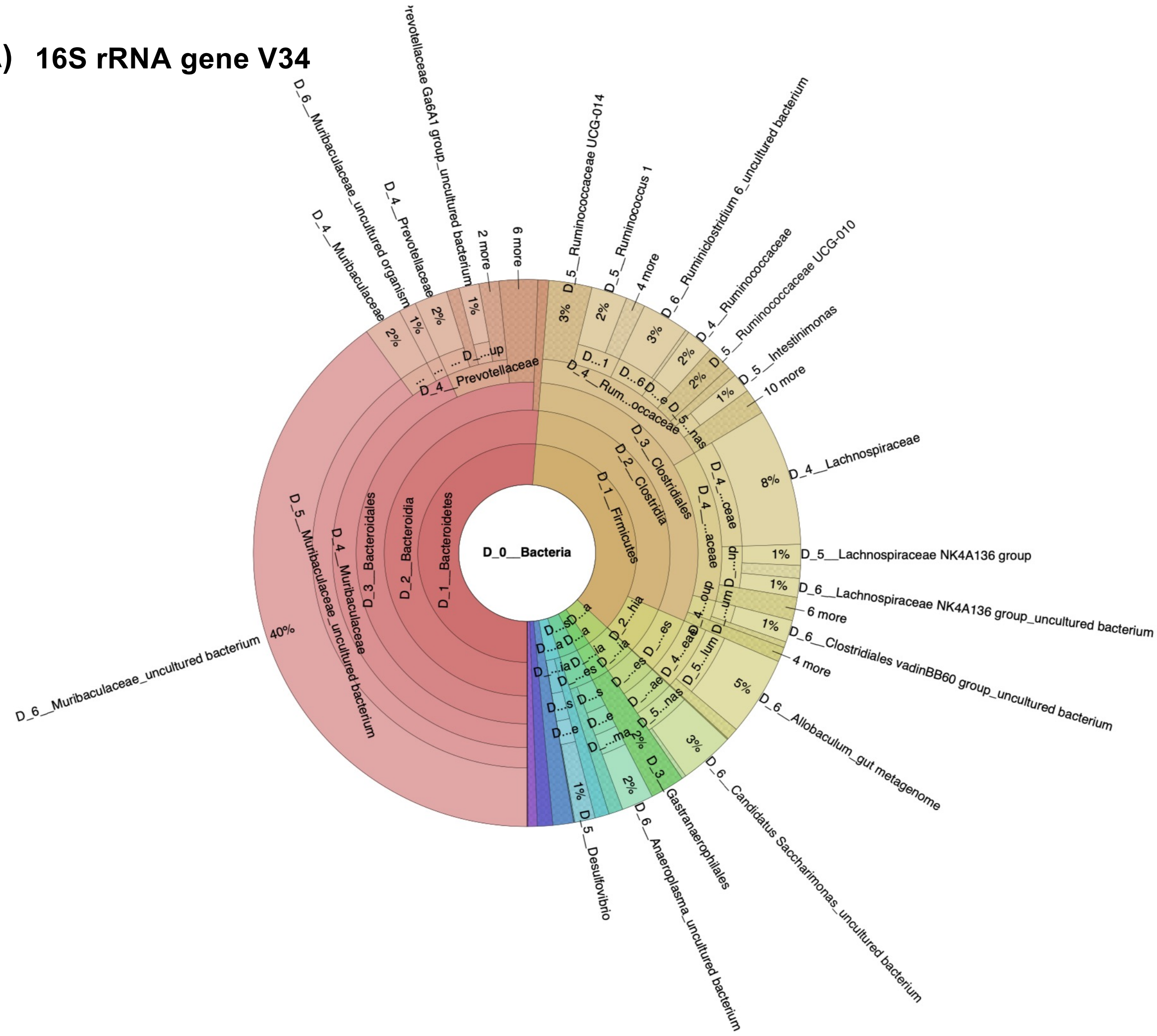

B) WGS

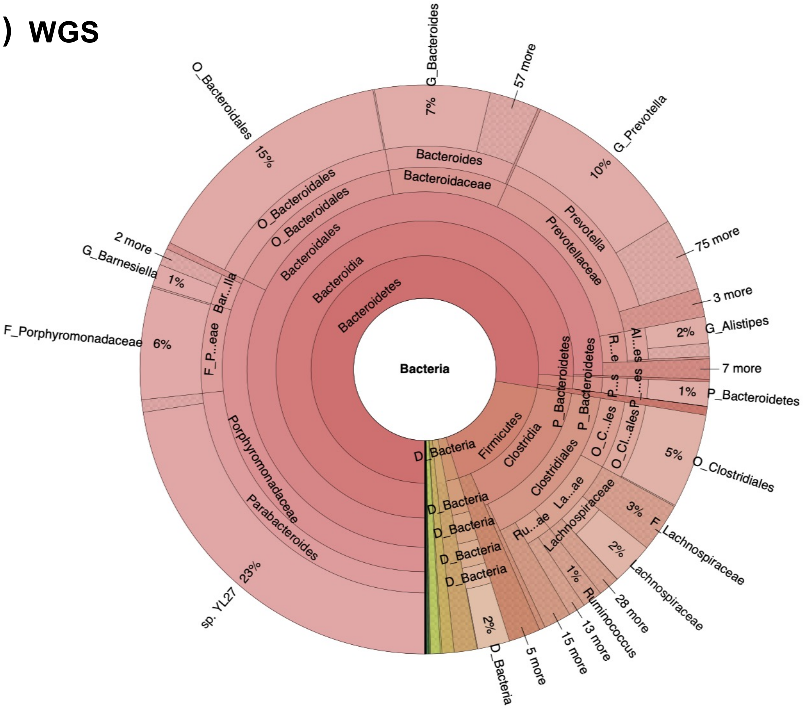

Figure S6. Comparison Krona plots of 16S- and WGS-detected taxa in females post-live-*L. reuteri* treatment.
Krona plots comparing relative abundance of taxa averaged across 8 live-*L. reuteri*-treated females measured by **A)** 16S rRNA gene V34 amplicon, and **B)** Whole-genome shotgun sequence.
