# Supplementary material for: Limosilactobacillus reuteri administration alters the gut-brain-behavior axis in a sex-dependent manner in socially monogamous prairie voles
Source: Front Microbiol. 2023 Feb 8;14:1015666. doi: 10.3389/fmicb.2023.1015666 (PMC9945313; doi:10.3389/fmicb.2023.1015666)
Supplement: Supplementary file 1 [file Data_Sheet_1.zip › Tables S1 and S2.docx]

**Table S1** Average changes (post-pre) in weight and average water intake in prairie voles.

*: Mean ± SEM

| Group | HK *L. reuteri* | | Live *L. reuteri* | | | |  | *p* |  |
| --- | --- | --- | --- | --- | --- | --- | --- | --- | --- |
| **Sex** | **M** | **F** | **M** | | **F** | **Group** | | ***Sex*** | ***GxS*** |
|  |  |  |  | | | |  |  |  |
| **ΔWeight** | -1.00±0.50 | -2.08±0.33 | -1.13±0.48 | | -0.63±0.17 | | ns | ns | ns |
| Average water intake | 41.29±3.46^a^ | 28.67±2.64^b^ | | 29.09±1.31^b^ | 32.72±2.12^b^ | | ns | ns | < .01 |

Weight change was calculated by post-weight minus pre-weight (grams). Means with different letters are significantly different from each other.

**Table S2**. Additional cage and behavior durations (seconds) in social affiliation tests in male and female voles.

| Group | HK-*L. reuteri* | | | Live *L. reuteri* | | | |  | *p* |  |
| --- | --- | --- | --- | --- | --- | --- | --- | --- | --- | --- |
| **Sex** | **M** | | **F** | **M** | | **F** | **Group** | | ***Sex*** | ***GxS*** |
| **Stimulus Cage** | 631.3±132.7 | | 897.4±45.4 | 630.9±122.2 | 605.9±94.8 | | | ns | ns | ns |
| **Empty cage** | 506.2±119.6 | | 254.0±38.7 | 517.8±114.4 | 513.0±80.3 | | | ns | ns | ns |
| **Aggression** | 7.53±3.3 | | 5.81±4.6 | 6.56±1.8 | | 3.47±2.8 | | ns | ns | ns |
| **Corner sitting** | 4.50±1.9^ab^ | 9.81±2.5^b^ | | 1.20±0.5^a^ | 15.53±2.6^c^ | | | ns | **< .01** | **< .05** |
| **Digging** | 10.67±5.7 | 0.52±0.4 | | 16.24±8.9 | 0.28±0.3 | | | ns | **<.05** | ns |
| Drinking water | 0.54±0.3* | 2.04±1.8 | | 4.33±2.1 | 2.92±2.9 | | | ns | ns | ns |

*: Mean ± SEM
